# Identification of gastric cancer subtypes based on pathway clustering

**DOI:** 10.1038/s41698-021-00186-z

**Published:** 2021-06-02

**Authors:** Lin Li, Xiaosheng Wang

**Affiliations:** 1 Biomedical Informatics Research Lab, School of Basic Medicine and Clinical Pharmacy, China Pharmaceutical University, Nanjing, China; 2 Cancer Genomics Research Center, School of Basic Medicine and Clinical Pharmacy, China Pharmaceutical University, Nanjing, China; 3 Big Data Research Institute, China Pharmaceutical University, Nanjing, China

**Keywords:** Cancer genomics, Cancer microenvironment, Tumour immunology

## Abstract

Gastric cancer (GC) is highly heterogeneous in the stromal and immune microenvironment, genome instability (GI), and oncogenic signatures. However, a classification of GC by combining these features remains lacking. Using the consensus clustering algorithm, we clustered GCs based on the activities of 15 pathways associated with immune, DNA repair, oncogenic, and stromal signatures in three GC datasets. We identified three GC subtypes: immunity-deprived (ImD), stroma-enriched (StE), and immunity-enriched (ImE). ImD showed low immune infiltration, high DNA damage repair activity, high tumor aneuploidy level, high intratumor heterogeneity (ITH), and frequent *TP53* mutations. StE displayed high stromal signatures, low DNA damage repair activity, genomic stability, low ITH, and poor prognosis. ImE had strong immune infiltration, high DNA damage repair activity, high tumor mutation burden, prevalence of microsatellite instability, frequent *ARID1A* mutations, elevated *PD-L1* expression, and favorable prognosis. Based on the expression levels of four genes (*TAP2*, *SERPINB5*, *LTBP1*, and *LAMC1*) in immune, DNA repair, oncogenic, and stromal pathways, we developed a prognostic model (IDOScore). The IDOScore was an adverse prognostic factor and correlated inversely with immunotherapy response in cancer. Our identification of new GC subtypes provides novel insights into tumor biology and has potential clinical implications for the management of GCs.

## Introduction

Gastric cancer (GC) is the second leading cause of cancer deaths in the world^1^ and particularly prevails in East Asia^2^. Abundant evidence indicates that GC is highly heterogeneous^3^. Based on the pathohistological classification, GC includes following three subtypes: intestinal, diffuse, and indeterminate^4^. Based on molecular profiles, GC includes four subtypes defined by The Cancer Genome Atlas (TCGA): Epstein–Barr virus (EBV) associated, microsatellite instable (MSI), genomically stable (GS), and chromosomal instability (CIN)^5^. In addition, the four molecular subtypes defined by the Asian Cancer Research Group (ACRG), include microsatellite stable (MSS)/epithelial–mesenchymal transition (EMT), MSI, MSS/p53+, and MSS/p53−^6^. The high heterogeneity in GC brings great challenges to the successful treatment of this disease^3^. Traditional treatment strategies, including surgery, chemotherapy, and radiotherapy, often have limited efficacy for the refractory or metastatic GCs^7^. Targeted therapies for GC, such as targeting HER2, EGFR, FGFR, KIT, c-Met, VEGFR, and CLDN18.2, are currently under investigation, although most targeted therapies demonstrated moderate effect or drug resistance^8^.

Cancer immunotherapies, such as immune checkpoint inhibitors (ICIs)^9^, have achieved success in treating various refractory malignancies, including the MSI subtype of GC. Nevertheless, only a subset of cancer patients displayed a favorable response to immunotherapies. To improve the immunotherapeutic efficiency, the discovery of predictive biomarkers for immunotherapy response is crucial. Some such biomarkers have been identified, including PD-L1 expression^10^, DNA mismatch repair deficiency or MSI^11^, and tumor mutation burden (TMB)^12^. Besides, the “hot” tumors with high immune infiltration often display a more active response to immunotherapy than the “cold” tumors with inferior immune infiltration^13^. Thus, the identification of actionable targets for intervention to enhance tumor immune infiltration is significant. Several studies have investigated the molecular characteristics associated with tumor immunity in GC^14–16^. Our recent study revealed that *TP53* mutations correlated with suppressive antitumor immunity in GC by immunogenomics analysis^14^. Park et al.^15^ developed immune gene signatures to classify GC patients into three immune subtypes, which had significantly different prognoses. Zeng et al.^16^ defined three GC subtypes based on immune cell infiltration patterns in the tumor microenvironment (TME).

Despite these various molecular classification methods for GC^5,6,15,16^, a combination of immune pathways and other GC-associated pathways for classifying GC remains lacking. Because GC is heterogenous in the immune microenvironment^15^, stromal microenvironment^17^, genome integrity^5^, and oncogenic signatures^18^, a classification of GC based on these features may provide new insights into the heterogeneity in GC. To this end, we performed clustering analysis of GCs based on the enrichment levels of four types of pathways, including immune pathways (natural killer cell-mediated cytotoxicity, antigen processing and presentation, T cell receptor signaling, B cell receptor signaling, and Fc gamma R-mediated phagocytosis), stromal pathways (ECM–receptor interaction, focal adhesion, and tight junction), DNA damage repair pathways (p53 signaling, mismatch repair, and homologous recombination), and oncogenic pathways (PI3K-Akt signaling, Wnt signaling, TGF-β signaling, and cell cycle). For each of the four pathway types, we selected several representative KEGG pathways. For example, among the immune pathways, the natural killer cell-mediated cytotoxicity pathway represents the innate immune response, the T cell receptor signaling pathway represents the adaptive immune response, and the antigen processing and presentation pathway is crucial for the presentation of tumor-specific antigens to T cells to eradicate tumor cells^19^. Among the stromal pathways, the ECM–receptor interaction pathway derives signals that are critically involved in the regulation of EMT to modulate various behaviors of the tumor cells and cancer‐associated stromal cells^20,21^. DNA damage repair is critical for maintaining genome integrity^22^. We selected its representative pathways: p53 signaling, mismatch repair, and homologous recombination. The p53 signaling pathway plays a key role in the DNA damage response^23^, while *TP53* mutations occur in around half of GCs^24^. Both mismatch repair and homologous recombination pathways are important for maintaining genome integrity, and their deficiency is the most common DNA damage repair deficiency in GC^5^. The deficiency of mismatch repair is responsible for MSI, a type of small-scale genomic instability displayed in about 20% of GCs^25^. In contrast, the deficiency of homologous recombination is responsible for CIN, a type of large-scale genomic instability shown in about 50% of GCs^5^. Although there are numerous oncogenic pathways associated with GC, we selected the four oncogenic pathways from the KEGG pathway map of “Gastric cancer” (https://www.kegg.jp/kegg-bin/show_pathway?hsa05226). We evaluated the enrichment levels of pathways using the single-sample gene-set enrichment analysis (ssGSEA) scores^26^. The ssGSEA scores-based clustering method is more robust than the gene expression values-based method for identifying cancer subtypes and has been widely used for clustering analysis^27–29^. Based on the four types of pathways, we identified three GC subtypes, which were consistently shown in three different datasets. We comprehensively characterized molecular and clinical features associated with these subtypes. Our novel classification method may provide new insights into tumor biology as well as clinical implications for GC diagnosis and treatment.

## Results

### Pathway clustering identifies three GC subtypes

**Fig. 1:**
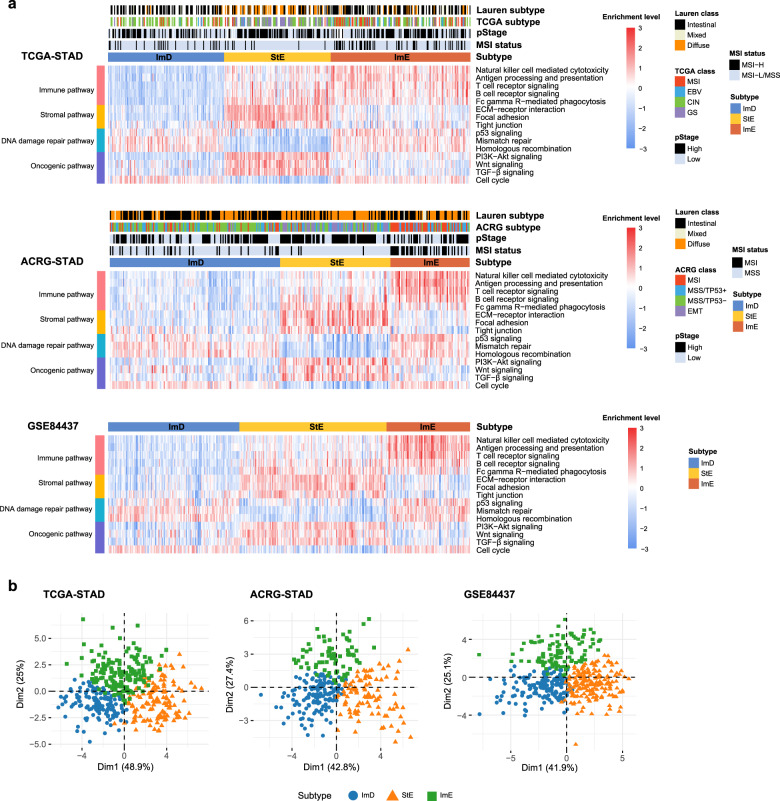
Identification of subtypes of gastric cancer based on pathway clustering. **a** Consensus clustering of gastric cancer (GC) identifies three subtypes (ImD, StE, and ImE) based on the enrichment levels of 15 pathways in 3 different datasets (TCGA-STAD, ACRG-STAD, and GSE84437). The enrichment levels of the pathways were evaluated by ssGSEA^26^ of all genes involved in them. The 15 pathways are associated with immune, DNA damage repair, oncogenic, and stromal signatures. **b** PCA confirms that GCs can be clearly separated into three subgroups based on the ssGSEA scores of the pathways. ImD immunity-deprived, StE stroma-enriched, ImE immunity-enriched, MSI microsatellite instable, MSS microsatellite stable, MSI-H high microsatellite instability, MSI-L low microsatellite instability, EMT epithelial–mesenchymal transition (These also apply to the following figures).

Based on the enrichment levels of 15 pathways, which were immune, stromal, DNA damage repair, or oncogenic pathways, we clustered GCs in three datasets (TCGA-STAD, ACRG-STAD, and GSE84437), respectively, using the consensus clustering algorithm^30^. Interestingly, all three datasets displayed similar clustering results, with GCs being clearly divided into three subtypes, termed immunity-deprived (ImD), stroma-enriched (StE), and immunity-enriched (ImE) (Fig. 1a). Principal component analysis confirmed that GCs could be clearly separated into three subgroups based on the pathway scores in all three datasets (Fig. 1b). ImD highly expressed the pathways of DNA damage repair and cell cycle, while it lowly expressed the immune, stromal, and other oncogenic (PI3K-Akt, Wnt, and TGF-β) pathways. In contrast, StE was characterized by the high enrichment of the stromal, PI3K-Akt, Wnt, and TGF-β pathways, and the low enrichment of the DNA damage repair and cell cycle pathways. ImE presented elevated activities of the immune, DNA damage repair, and cell cycle pathways and reduced activities of the stromal, PI3K-Akt, Wnt, and TGF-β signaling pathways.

### Immune and stromal signatures and tumor purity of the GC subtypes

**Fig. 2:**
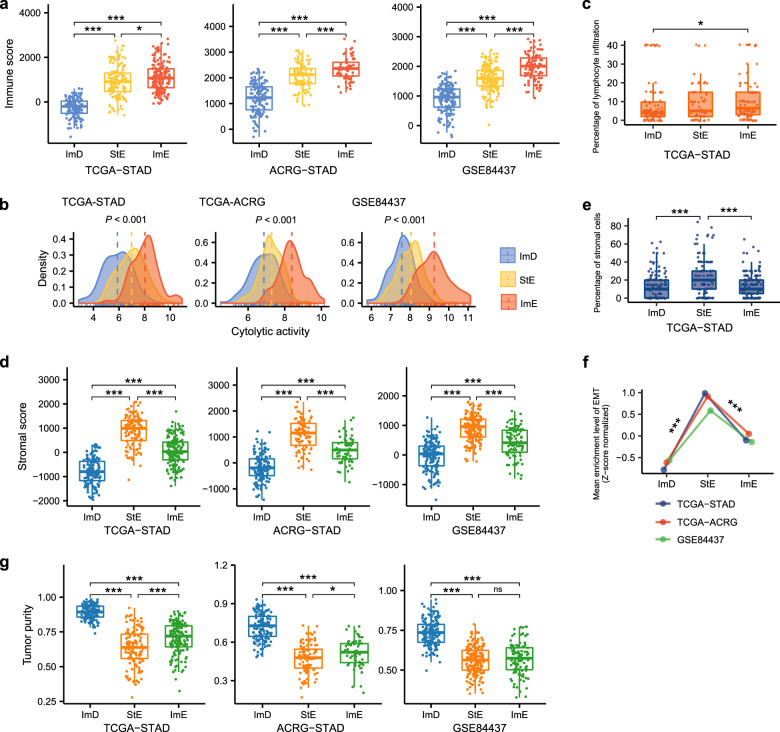
Comparisons of immune and stromal signatures and tumor purity between the three GC subtypes. The immune scores (**a**), cytolytic activity (**b**), and percentages of lymphocyte infiltration (**c**) are the highest in ImE and the lowest in ImD. The stomal scores (**d**), percentages of stromal cells (**e**), and activity of EMT (**f**) are the highest in StE and the lowest in ImD. **g** ImD has the highest tumor purity, and StE has the lowest tumor purity. The immune and stomal scores and tumor purity were evaluated by ESTIMATE^31^. The cytolytic activity is the average expression level of two marker genes (*GZMA* and *PRF1*)^32^. The activity of EMT is the ssGSEA score^26^ of its marker genes. The one-tailed Mann–Whitney *U* test *P* values are indicated. *P < 0.05, **P < 0.01, ***P < 0.001.

We compared immune scores, immune cytolytic activity, percentages of lymphocyte infiltration, stromal scores, percentages of stromal cells, activity of EMT, and tumor purity between the three GC subtypes. The immune and stromal scores were calculated by the ESTIMATE algorithm^31^ based on gene expression profiles of immune signature and stromal signature in the tumor, respectively. We also used ESTIMATE to evaluate tumor purity, which is a cosine function of the sum of immune and stromal scores^31^. The immune cytolytic activity represents the ability of cytotoxic T cells and natural killer cells to eliminate tumor cells, which was the average expression level of two marker genes (*GZMA* and *PRF1*) in the tumor^32^. We obtained percentages of lymphocyte infiltration and stromal cells from the TCGA GC pathological slides data (https://portal.gdc.cancer.gov/). The three GC subtypes had significantly different immune scores: ImD < StE < ImE, in all three datasets (one-tailed Mann–Whitney *U* test, *P* < 0.015) (Fig. 2a). The expression levels of most human leukocyte antigen (HLA) genes showed the pattern: ImD < StE < ImE (one-way ANOVA test, *P* < 0.001) (Supplementary Fig. 1a). The immune cytolytic activity was the highest in ImE and the lowest in ImD (*P* < 0.001) (Fig. 2b). These data confirmed that ImE and ImD had the highest and lowest antitumor immunity, respectively. We further verified this result with the TCGA GC pathological slides data, which showed that ImE had higher percentages of lymphocyte infiltration than ImD (*P* = 0.043) (Fig. 2c). In addition, we evaluated the proportions of 22 immune cells in the GC subtypes using the CIBERSORT algorithm^33^. We found that StE had significantly higher proportions of resting CD4 memory T cells, resting mast cells, and immune-inhibitory M2 macrophages but lower proportions of activated CD4 memory T cells and immune-stimulatory M1 macrophages than ImE (*P* < 0.01) (Supplementary Fig. 1b). Moreover, StE had significantly higher proportions of resting mast cells and M2 macrophages but lower proportions of activated mast cells than ImD (*P* < 0.01). Furthermore, the ratios of immune-stimulatory over immune-inhibitory signatures (pro-/anti-inflammatory cytokines and M1/M2 macrophages) were significantly lower in StE than ImE and ImD (Supplementary Fig. 1c). These results indicate that StE is likely to display stronger immunosuppressive signatures than the other subtypes.

The stomal scores were significantly different between the three GC subtypes: ImD < ImE < StE (*P* < 0.001) (Fig. 2d), confirming that StE had the strongest stromal signatures among the three subtypes. The TCGA GC pathological slides data also showed that StE had significantly higher percentages of stromal cells than ImD and ImE (*P* < 0.001) (Fig. 2e). The activation of the EMT biological process may alter the TME to activate stromal signatures^34^. As expected, the activity of EMT was significantly higher in StE than ImD and ImE (*P* < 0.001) (Fig. 2f). Furthermore, we compared the expression levels of 194 stromal gene signatures^35^ between the three GC subtypes and found that most of them were more highly expressed in StE than ImD and ImE (two-tailed student’s *t* test, *P* < 0.05) (Supplementary Fig. 1d). In contrast, tumor purity displayed an opposite trend: ImD > ImE > StE (*P* < 0.05) (Fig. 2g), indicating that ImD and StE had the highest and lowest tumor purity, respectively. To correct for the impact of tumor purity on the associations of GC subtypes with an immune score, stromal score, and EMT signature, we built logistic regression models with three predictors (StE, ImE, and tumor purity) to predict the immune score, stromal score, and EMT signature score in the three datasets. We found that both StE and ImE were significant positive predictors for the immune score. Meanwhile, StE was a significant positive predictor for stromal score and EMT signature (*P* < 0.05) (Supplementary Fig. 2). These results suggest that the significant associations of the GC subtypes with immune and stromal signatures are independent of tumor purity.

### Genomic features of the GC subtypes

**Fig. 3:**
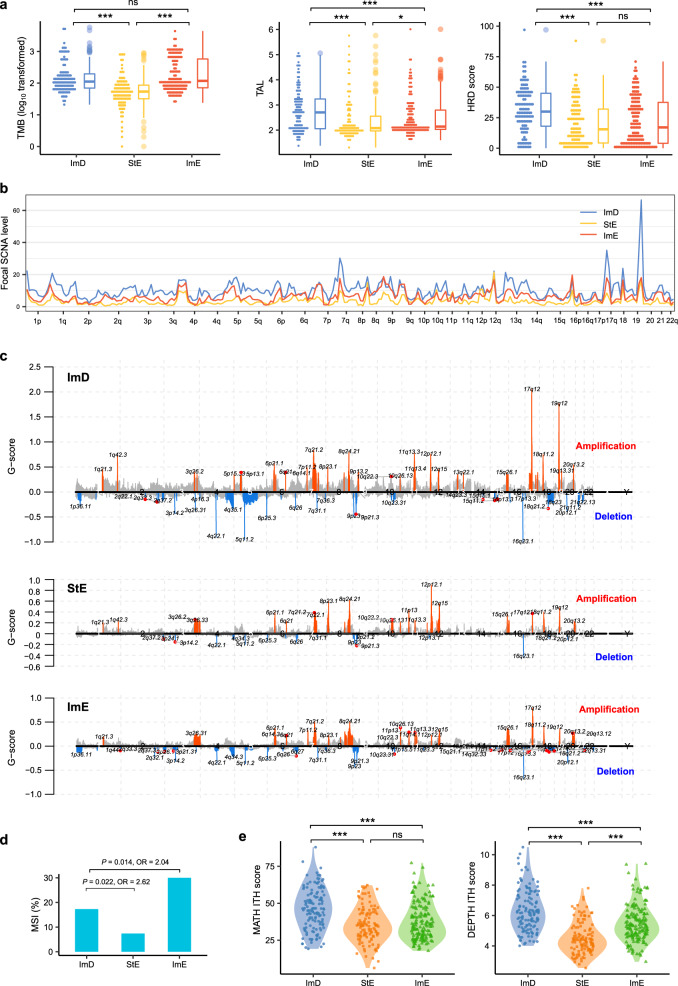
Comparisons of genome instability and intratumor heterogeneity between the three GC subtypes in TCGA-STAD. **a** Comparisons of TMB, TAL, and HRD scores between the three GC subtypes. **b**, **c** ImD and StE have the highest and lowest levels of SCNAs, respectively. The SCNA levels and *G*-scores were calculated by GISTIC2^98^. **d** ImE and StE harbor the highest and lowest proportion of MSI cancers, respectively. The Fisher’s exact test *P* values and odds ratios are shown. **e** ImD and StE display the highest and lowest ITH, respectively. The one-tailed Mann–Whitney *U* test *P* values are indicated in (**a**) and (**e**). The MATH^96^ and DEPTH^97^ algorithms were used to evaluate ITH at the DNA and mRNA levels, respectively. TMB tumor mutation burden, TAL tumor aneuploidy level, HRD homologous recombination deficiency, SCNAs somatic copy number alterations, OR odds ratio, ITH intratumor heterogeneity, ns, not significant, *P < 0.05, **P < 0.01, ***P < 0.001.

Genomic instability (GI) plays a key role in tumor initiation and progression^36^. GI includes small-scale GI leading to increased TMB and large-scale GI leading to increased tumor aneuploidy level (TAL)^37^. We found that TMB was significantly higher in ImD and ImE than StE in TCGA-STAD (*P* < 0.001), while it showed no significant difference between ImD and ImE (*P* = 0.42) (Fig. 3a). Because MSI tumors have high TMB and were the most prevalent in ImE, we compared TMB between the three subtypes with MSI tumors excluded. We found that TMB was still significantly higher in ImD and ImE than StE in TCGA-STAD (*P* < 0.001) and had no significant difference between ImD and ImE (*P* = 0.09). Similarly, TAL was significantly higher in ImD and ImE than StE (*P* ≤ 0.01) (Fig. 3a). Moreover, ImD displayed significantly higher TAL than ImE (*P* < 0.001). Homologous recombination deficiency (HRD) may cause large-scale GI^38^. We found that HRD scores were significantly higher in ImD than StE and ImE (Fig. 3a). We further compared somatic copy number alteration (SCNA) levels between the three subtypes. As expected, ImD displayed significantly higher levels of arm- and focal-level SCNAs than StE and ImE (*P* < 0.001 for comparisons of amplification and deletion in arm-level SCNAs and total alterations in focal-level SCNAs) (Fig. 3b, c, Supplementary Fig. 3); ImE tended to have higher levels of arm- and focal-level SCNAs than StE. In addition, ImE harbored a significantly higher proportion of MSI cancers than ImD, which in turn harbored a significantly higher proportion of MSI cancers than StE (Fisher’s exact test, *P* < 0.05, odds ratio (OR) > 2) (Fig. 3d). Collectively, these data indicate that ImD and ImE are more genomically instable than StE, while ImD and ImE are characterized by large-scale and small-scale GI, respectively. GI often causes intratumor heterogeneity (ITH), which is genetic and phenotypic variation within tumors and is associated with tumor progression, immune evasion, and drug resistance^39^. As expected, ImD and ImE tended to display higher ITH than StE (Fig. 3e). Meanwhile, ImD had higher ITH than ImE (*P* < 0.001), suggesting that large-scale GI is likely to cause higher ITH than small-scale GI.

### Mutation profiles of the GC subtypes

**Fig. 4:**
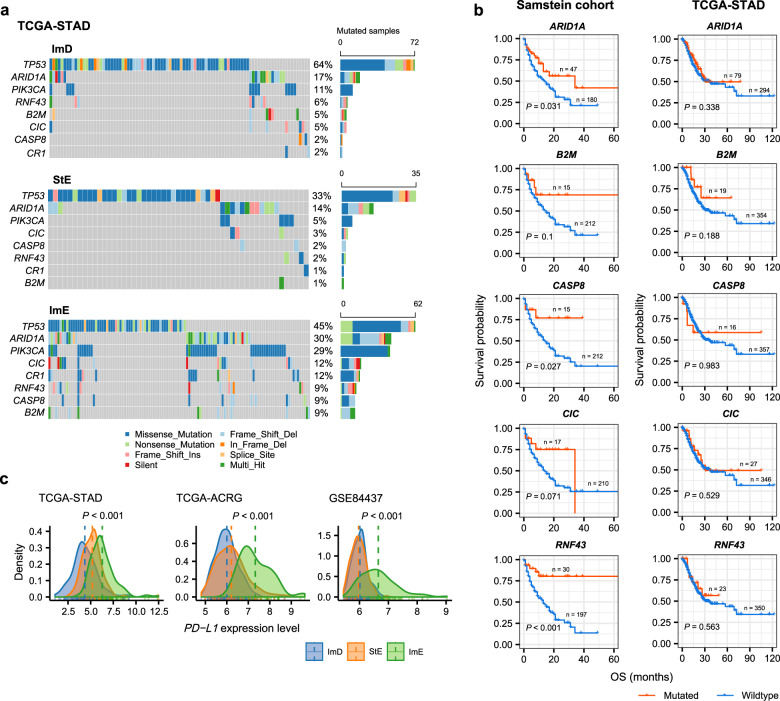
Comparisons of mutation profiles between the three GC subtypes. **a** Eight genes showing significantly different mutation frequencies between the three GC subtypes in TCGA-STAD. **b** Five genes more frequently mutated in ImE than ImD and StE, whose mutations are correlated with better OS in the Samstein cohort (gastrointestinal cancer)^46^ receiving immune checkpoint inhibitor treatment (log-rank test, *P* ≤ 0.1), but have no a significant correlation with OS in TCGA-STAD without such treatment. Kaplan–Meier curves are used to compare the survival time, and the log-rank test *P* values are shown. OS overall survival. **c** Comparisons of *PD-L1* expression levels between the three GC subtypes. The one-way ANOVA test *P* values are shown.

The mutation of cancer driver genes may affect various key cellular functions to drive cancer development^40^. We compared the mutation frequencies of 172 driver genes^41^ between the three GC subtypes in TCGA-STAD (Supplementary Table 1). Notably, ImD displayed a significantly higher mutation rate of *TP53* than StE and ImE (*P* < 0.01, OR > 2), and ImE had a higher *TP53* mutation rate than StE (*P* = 0.086, OR = 1.6) (Fig. 4a). These results conform to the significant difference in GI between the three subtypes since p53 plays a prominent role in the maintenance of genomic stability^42^. *ARID1A*, a component of the ATP-dependent chromatin remodeling complex SNF/SWI, was more frequently mutated in ImE than ImD and StE (*P* < 0.02, OR > 2) (Fig. 4a). This is consistent with the significant positive association between *ARID1A* mutations and MSI in gastrointestinal cancers^43^ since ImE harbored a significantly higher proportion of MSI cancers than the other subtypes. *PIK3CA*, *CASP8*, and *CR1* were also more frequently mutated in ImE than ImD and StE (*P* < 0.03, OR > 3) (Fig. 4a). Previous studies have demonstrated the associations of *PIK3CA* mutations^44^ and *CASP8*^45^ mutations with increased immune infiltration in cancer, consistent with the highly enriched anti-tumor immune signatures in ImE vs. ImD and StE. In addition, numerous genes showed higher mutation rates in ImE and/or ImD than StE, including *ZBTB20*, *CSMD1*, *HLA-B*, *DCLK1*, *NWD1*, *DMXL2*, *EAF2*, *ERBB3*, *CNTLN*, *OR2G6*, *BCLAF1*, *PLEKHA6*, and *ZNF676* (*P* < 0.1, OR > 2) (Supplementary Fig. 4). Interestingly, we found that the mutations of several genes (*ARID1A*, *B2M*, *CASP8*, *CIC*, and *RNF43*), which had significantly higher mutation rates in ImE than ImD and StE, were associated with better overall survival (OS) in the Samstein cohort (gastrointestinal cancer)^46^ treated with ICIs (log-rank test, *P* ≤ 0.1) but showed no significant correlation with OS in TCGA-STAD not treated with ICIs (Fig. 4b). These results indicate that ImE tumors are more likely to respond to ICIs than the other subtypes. It is justified because ImE has the highest immune infiltration (Fig. 1a) and PD-L1 expression levels (one-way ANOVA test, *P* < 0.001) (Fig. 4c), both of which are the determinants driving the response to ICIs^47^.

### DNA methylation profiles of the GC subtypes

**Fig. 5:**
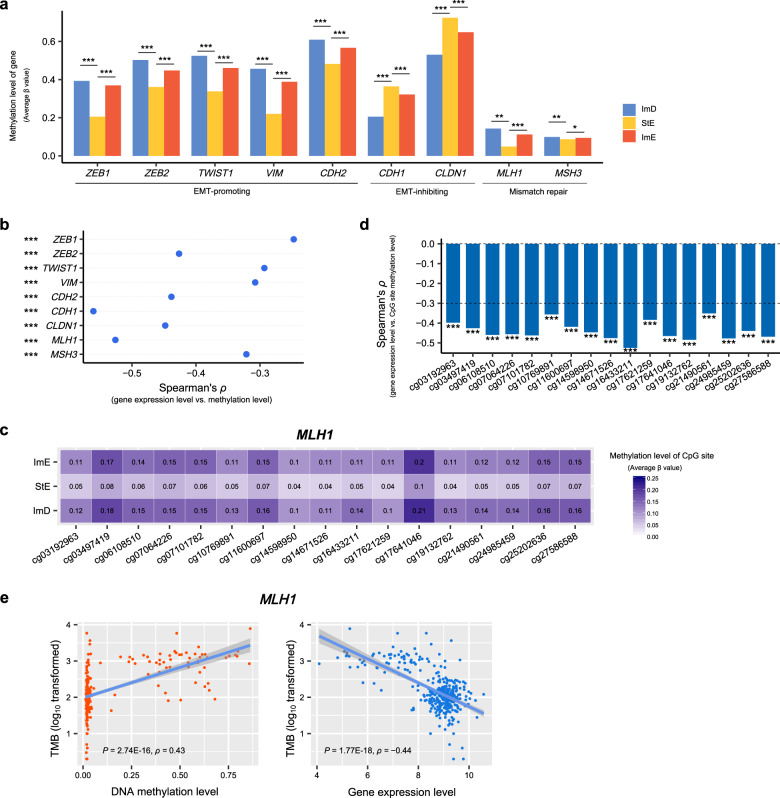
Comparisons of DNA methylation profiles between the three GC subtypes in TCGA-STAD. **a** The EMT-promoting, EMT-inhibiting, and DNA mismatch repair genes displaying significantly different methylation levels between the three GC subtypes. The one-tailed Mann–Whitney *U* test *P* values are indicated. **b** Correlations between expression levels and methylation levels of the genes whose methylation levels are significantly different between the three GC subtypes. **c** 17 CpG sites within *MLH1* CpG islands having significantly lower methylation levels in StE than ImD and ImE. The methylation levels (average β values) are shown. **d** Spearman correlations between *MLH1* expression levels and the methylation levels of its 17 CpG sites, which have significantly lower methylation levels in StE than ImD and ImE. **e** Spearman correlations between TMB and *MLH1* methylation levels and expression levels. *P < 0.05, **P < 0.01, ***P < 0.001.

DNA methylation alterations in tumorigenesis are well recognized^48^. We found several EMT-promoting genes^49–51^ showing significantly lower methylation levels in StE than ImD and ImE in TCGA-STAD (Fig. 5a). These genes included *ZEB1*, *ZEB2*, *TWIST1*, *VIM*, and *CDH2*. In contrast, *CDH1* and *CLDN1*, which play a role in repressing EMT, had significantly higher methylation levels in StE than ImD and ImE. These results reflect that the EMT promoters are upregulated in StE, while the EMT repressors are downregulated in this subtype. It is consistent with the fact that StE has the strongest EMT signature among the three GC subtypes. Several DNA repair genes, including *MLH1* and *MSH3*, displayed significantly lower methylation levels in StE than ImD and ImE (Fig. 5a). As expected, the expression levels of both genes were inversely correlated with their methylation levels in TCGA-STAD (Spearman’s correlation *ρ* < −0.2, *P* < 0.001) (Fig. 5b). Furthermore, we found that 17 CpG sites within *MLH1* CpG islands had significantly lower methylation levels in StE than ImD and ImE (Fig. 5c). Also, the methylation levels of these CpG sites had significant negative correlations with the expression levels of *MLH1* in TCGA-STAD (*ρ* < −0.3, *P* < 0.001) (Fig. 5d). These results indicate that StE has a stronger DNA repair function to maintain its genomic stability (such as low TMB) compared to the other subtypes. Indeed, TMB displayed a significant positive correlation with the methylation levels of *MLH1* (*ρ* = 0.43, *P* = 2.74 × 10^−16^) and a significant negative correlation with the expression levels of *MLH1* (*ρ* = −0.44, *P* = 1.77 × 10^−18^) in TCGA-STAD (Fig. 5e).

### Protein expression profiles of the GC subtypes

**Fig. 6:**
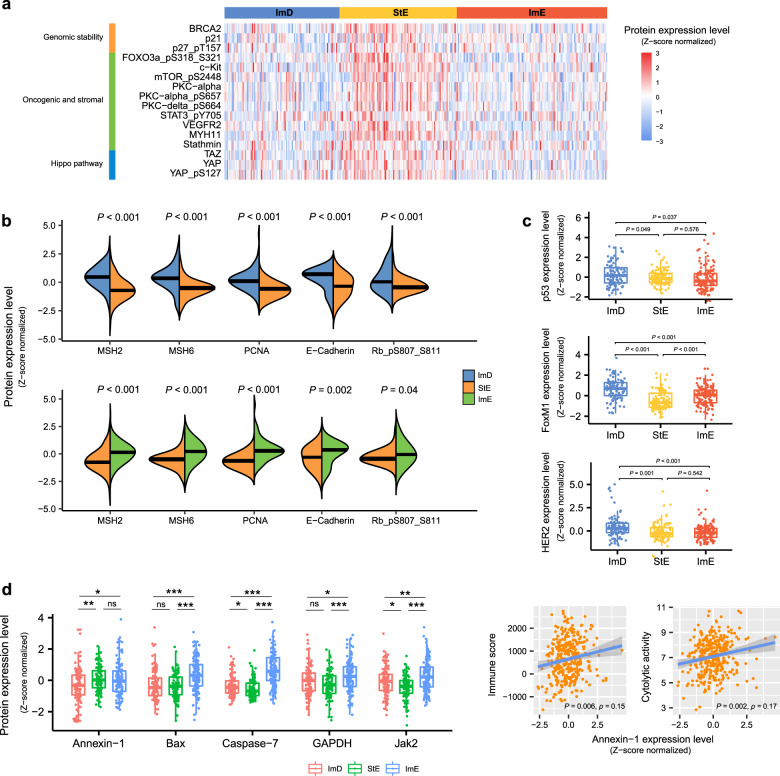
Comparisons of protein expression profiles between the three GC subtypes in TCGA-STAD. **a** Heatmap showing that the proteins maintaining genomic stability, correlating with oncogenic and stromal signatures, and regulating the Hippo pathway display significantly higher expression levels in StE than ImD and ImE (two-tailed Student’s *t* test, false discovery rate < 0.05). **b** The DNA repair, cellular adhesion, and tumor suppression proteins displaying significantly lower expression levels in StE than ImD and ImE. **c**, **d** Comparisons of the expression levels of p53, FoxM1, HER2, Annexin-1, Bax, Caspase-7, GAPDH, and Jak2 between the three GC subtypes, and Spearman correlations between Annexin-1 expression levels and immune signature scores. The two-tailed Student’s *t* test *P* values are indicated in (**b**–**d**). *P < 0.05, **P < 0.01, ***P < 0.001.

Based on the TCGA protein expression profiling data, we analyzed the expression levels of 219 proteins in the GC subtypes (Supplementary Table 2). We found that several proteins functioning in the maintenance of genomic stability had significantly higher expression levels in StE than ImD and ImE (two-tailed Student’s *t* test, false discovery rate (FDR) < 0.05) (Fig. 6a). These proteins included BRCA2, p21, and p27_pT157. This may explain why StE is more GS than ImD and ImE. Besides, many oncogenic and stromal proteins were more highly expressed in StE than ImD and ImE, including FOXO3a_pS318_S321, c-Kit, mTOR_pS2448, PKC-alpha, PKC-alpha_pS657, PKC-delta_pS664, STAT3_pY705, VEGFR2, MYH11, and Stathmin (Fig. 6a). Several proteins regulating the Hippo pathway also showed significantly higher expression levels in StE than ImD and ImE, such as TAZ, YAP, and YAP_pS127, consistent with the roles of the Hippo pathway in promoting stromal signatures^52^ and protecting genomic stability^53^. In contrast, several DNA repair proteins displayed significantly lower expression levels in StE than ImD and ImE, including MSH2, MSH6, and PCNA (Fig. 6b). Again, this is consistent with the higher genomic stability of StE relative to the other subtypes. E-cadherin was also significantly downregulated in StE vs. ImD and ImE, consistent with its role in promoting cellular adhesion and inhibiting cellular motility^54^. In addition, the tumor suppressor protein Rb_pS807_S811 was more lowly expressed in StE vs. ImD and ImE. This could lead to poorer clinical outcomes in StE. Interestingly, we found that p53, a key maintainer of genomic stability^42^, was more highly expressed in ImD than StE and ImE (Fig. 6c), whereas ImD was characterized by large-scale GI. Compensatory activation of p53 might explain this result since ImD had a significantly higher mutation rate of *TP53* than StE and ImE. FoxM1, a member of the FOX family of transcription factors, displayed higher expression levels in ImD vs. StE and ImE and in ImE vs. StE (Fig. 6c). This result conforms to the fact that FoxM1 upregulation can induce GI in cancer^55^. HER2 was also more highly expressed in ImD than StE and ImE (Fig. 6c). It indicates that HER2-amplified GCs are more likely to belong to the ImD subtype. In contrast, Annexin-1, an immunomodulatory protein playing diverse roles in cancer^56^, was more lowly expressed in ImD than StE and ImE (Fig. 6d). This result indicated a positive correlation between Annexin-1 expression and anti-tumor immune response in GC since ImD was immune-deprived. In fact, the expression levels of Annexin-1 were positively correlated with immune score and immune cytolytic activity in GC (Fig. 6d). It is consistent with the argument that increased expression of Annexin-1 during pathological conditions may drive hyperactivation of T cells^57^. Caspase-7, which is a member of the caspase family of proteins and plays a crucial role in inducing apoptosis^58^, was more highly expressed in ImE than ImD and StE (Fig. 6d). Another apoptosis-inducing protein Bax also showed significantly higher expression levels in ImE than ImD and StE (Fig. 6d). These results are consistent with the positive association between the apoptosis activity and antitumor immunity in cancer^14^. Besides, GAPDH, an enzyme catalyzing the sixth step of glycolysis, was more highly expressed in ImE than ImD and StE (Fig. 6d). This is accordant with our previous finding that increased glycolysis promotes anti-tumor immunity^59^. Jak2, a member of the Janus kinase family, was also more highly expressed in ImE than ImD and StE (Fig. 6d). Again, this is consistent with the stronger antitumor immune signature in ImE vs. ImD and StE since the JAK-STAT pathway involving Jak2 is a positive regulator of antitumor immune signature in cancer^14^.

### Clinical features of the GC subtypes

**Fig. 7:**
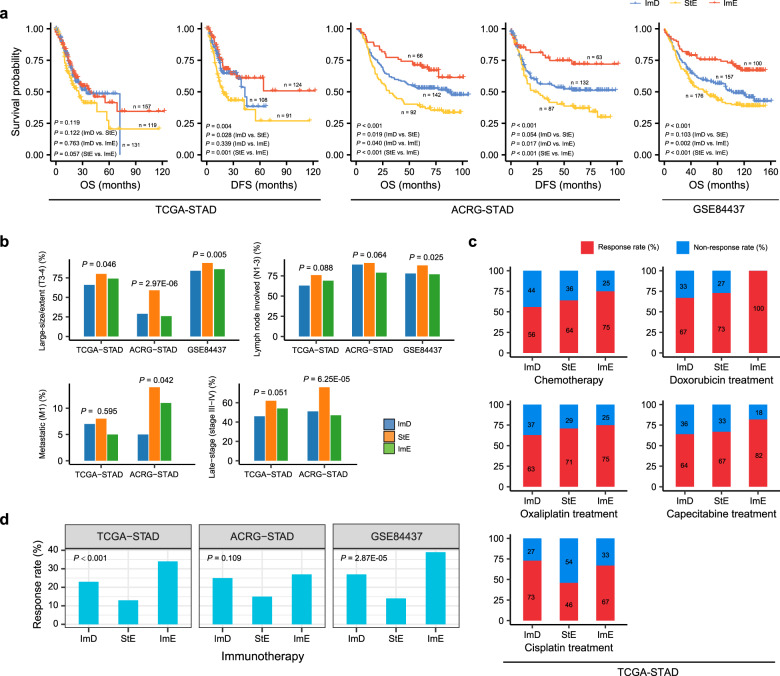
Comparisons of clinical features between the three GC subtypes. **a** Kaplan–Meier curves showing that ImE and StE tend to have the best and worst survival prognosis, respectively. The log-rank test *P* values are shown. DFS disease-free survival. **b** StE harbors a higher proportion of advanced (large size/extent (T3–4), lymph nodes involved (N1–3), metastatic (M1), or late-stage (stage III–IV)) tumors than ImD and ImE. The Fisher’s exact test *P* values are shown. **c** Comparisons of the response (complete or partial response) rates of chemotherapy (30 drugs combined) and four individual chemotherapies (doxorubicin, oxaliplatin, capecitabine, and cisplatin) between the GC subtypes. ImE showing the highest response rate of chemotherapy (combined), doxorubicin, oxaliplatin, and capecitabine; ImD showing the highest response rate to cisplatin. **d** ImE and StE having the highest and lowest response rates to immune checkpoint inhibitors, respectively, predicted by the TIDE algorithm^68^. Fisher’s exact test *P* values are shown.

Survival analyses showed that ImE tended to have the best survival (OS and disease-free survival (DFS)) prognosis, while StE had the worst survival among the three GC subtypes (Fig. 7a). The main reason behind this could be that ImE had the strongest antitumor immune response, while StE was the most enriched with stromal signatures. Actually, previous studies have demonstrated that tumor prognosis had a positive association with antitumor immune signatures^14,60,61^ and a negative association with stromal signatures^62–64^. Furthermore, we found that StE was likely to harbor a higher proportion of advanced tumors (large size/extent (T3–4), lymph nodes involved (N1–3), metastatic (M1), or late-stage (stage III–IV)) than ImD and ImE (Fig. 7b). For example, StE harbored 76% late-stage tumors vs. ImD (51%) and ImE (47%) in ACRG-STAD (StE vs. ImD: *P* < 0.001, OR = 3.1; StE vs. ImE: *P* < 0.001, OR = 3.6). In GSE84437, 94% tumors in StE had large size/extent vs. 84% in ImD and 86% in ImE (StE vs. ImD: *P* = 0.004, OR = 3.1; StE vs. ImE: *P* = 0.025, OR = 2.7). In addition, we compared the response rate of chemotherapy between the GC subtypes in TCGA-STAD. We found that the response (complete or partial response) rate of chemotherapy (30 drugs combined) followed the pattern: ImE (75%) > StE (64%) > ImD (56%), confirming the positive association between anti-tumor immune response and chemotherapy^65,66^. Moreover, we compared the response rate of four individual chemotherapies (doxorubicin, oxaliplatin, capecitabine, and cisplatin) between the GC subtypes (the other 26 drugs were not analyzed due to a small sample size associated with them). We found that almost all the drugs followed the same pattern: ImE > StE > ImD (Fig. 7c). An exception is a cisplatin, to which ImD had the highest response rate among the three subtypes (ImD: 73%; StE: 46%; ImE: 67%). A possible explanation is the high prevalence of HRD in ImD that promotes its sensitivity to cisplatin-based chemotherapy^67^. We further used the TIDE algorithm^68^ to predict the response to ICIs of the GCs in the three datasets. We found that the response rate followed the pattern: ImE > ImD > StE, consistently in the three GC datasets (Fig. 7d). These results indicate that ImE is likely to respond best to immunotherapy, while StE is likely to have the worst response.

### Gene ontology of the GC subtypes

**Fig. 8:**
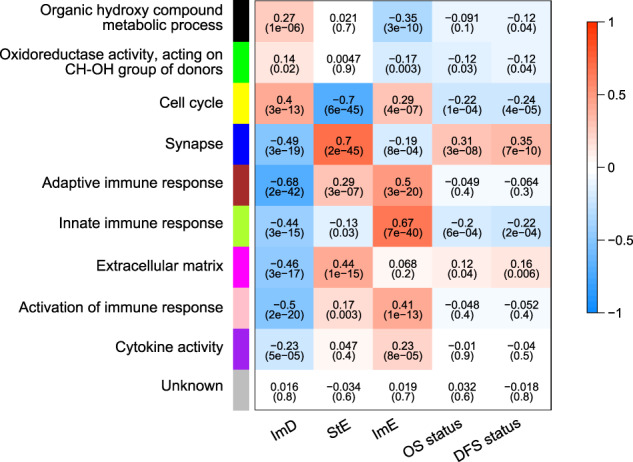
Nine gene modules significantly differentiating gastric cancers by the subtypes in ACRG-STAD. WGCNA^69^ showing that the immune responses are highly enriched in ImE and are deprived in ImD; the extracellular matrix is highly enriched in StE, and the cell cycle is downregulated in this subtype. Survival prognosis has positive correlations with the cell cycle and innate immune response and negative correlations with the synapse and extracellular matrix. The *P* values are shown in parenthesis.

We identified nine gene modules (gene ontology) significantly differentiating GCs by the subtypes in ACRG-STAD by using WGCNA^69^ (Fig. 8). As expected, the immune response (indicated by brown, green–yellow, pink, and purple colors) was highly enriched in ImE, while it was impoverished in ImD. The extracellular matrix, indicative of stromal signature, was highly enriched in StE, while the cell cycle was significantly downregulated in this subtype. In addition, the organic hydroxy compound metabolic process was significantly upregulated in ImD and downregulated in ImE. The blue gene module (synapse), which showed the strongest positive correlation with StE (*r* = 0.7), had significantly inverse correlations with OS and DFS prognosis. In contrast, the yellow gene module (cell cycle), with the strongest inverse correlation with StE (*r* = −0.7), was positively correlated with OS and DFS. In addition, the green–yellow gene module (innate immune response) had the strongest positive correlation with ImE (*r* = 0.67) and correlated positively with OS and DFS. These data are accordant with the previous results showing that StE and ImE had the worst and best survival prognosis among the three subtypes in ACRG-STAD. Overall, the gene ontology analysis recaptured the significantly different molecular and clinical characteristics between these GC subtypes.

### Oncogenic signatures of the GC subtypes

We found a number of oncogenic pathways whose activities were significantly higher in StE than ImD and ImE, including Wnt, mTOR, PI3K-Akt, JAK-STAT, RAS, MAPK, Hedgehog, Notch, HIF-1, TGF-β, and VEGF (Supplementary Table 3 and Supplementary Fig. 5). Among them, the Wnt, JAK-STAT, Hedgehog, and Notch signaling pathways play key roles in the regulation of development and stemness and have been associated with cancer^70–73^. A recent study showed that Wnt signaling was highly activated in the diffuse subtype of GC^74^, consistent with our result that the diffuse subtype was dominated by StE. The activation of Hedgehog signaling in tumor stroma has been shown to furnish a favorable microenvironment for tumor development^75,76^, supporting our result of the hyperactivation of Hedgehog signaling in StE. In addition, the PI3K/Akt/mTOR, RAS, and MAPK signaling pathways play important roles in driving tumor cell growth^77–79^, and the HIF-1, TGF-β, and VEGF pathways play crucial roles in promoting tumor progression and metastasis by modulating the TME^80–82^. Overall, the more active oncogenic signatures in StE may contribute to the worse clinical outcomes in this subtype, as has been shown in the previous results. Furthermore, all these pathways had significantly lower enrichment levels in ImD than ImE (Supplementary Fig. 5). These results indicate that inhibitors of these pathways are likely to be most effective for StE and least effective for ImD.

### Relationship between the pathway-based subtyping and other subtyping methods in GC

**Fig. 9:**
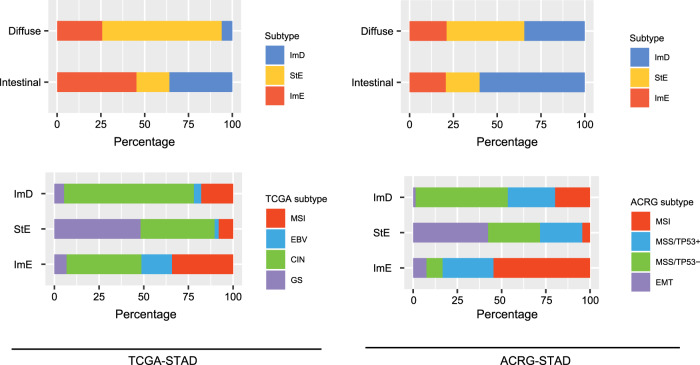
Comparisons between the pathway-based subtyping and other subtyping methods in GC. Intestinal and Diffuse are histological subtypes based on the pathohistological classification. The TCGA subtypes identified by integration of multi-omics data, including somatic mutations, SCNAs, CpG methylation, mRNA, miRNA, and protein expression. The ACRG subtypes identified based on the gene expression profiles of EMT, MSI, and *TP53* signatures. Intestinal is enriched with ImD and ImE, and Diffuse is dominated by StE. ImD contains CIN, StE contains GS and EMT, and ImE contains MSI and EBV-associated GCs, respectively. Chi-square test, *P* < 0.001.

We explored the relationship between our method and other GC subtyping methods^4–6^ We found that the intestinal histological subtype was dominated by ImD and ImE, while the diffuse subtype was dominated by StE (chi-square test, *P* < 0.001) (Fig. 9). It indicates that intestinal GCs could be either immune-inflamed or immune-deprived and that diffuse GCs are enriched with stromal signatures. In TCGA-STAD, MSI, and EBV-associated GCs were mainly included in ImE, CIN GCs were mostly included in ImD, and GS GCs were dominated by StE (*P* < 0.001). In addition, the EMT subtype of GCs in ACRG-STAD were mainly included in StE. These results are consistent with the characteristics of the pathway-based GC subtypes: ImE is immune-inflamed, ImD is immune-deprived due to chromosomal/genomic instability, and StE is GS and stromal signature enriched.

### Prediction of the pathway-based GC subtypes

**Fig. 10:**
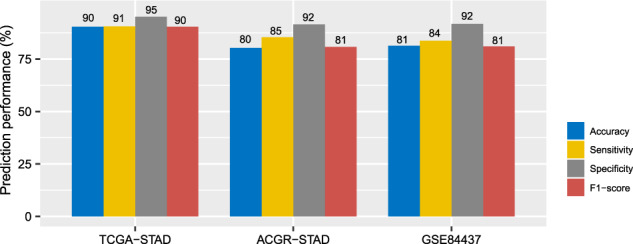
Prediction performance of the pathway-based GC classification method. TCGA-STAD as the training set and ACRG-STAD and GSE84437 as test sets to predict the three subtypes by XGBoost^83^. The prediction accuracies and weighted sensitivity, specificity, and F1-scores in TCGA-STAD (tenfold cross-validation), ACRG-STAD, and GSE84437 are shown.

To test the reproducibility and predictability of the pathway-based GC classification method, we used TCGA-STAD as the training set and ACRG-STAD and GSE84437 as test sets to predict the three subtypes with the XGBoost algorithm^83^. The tenfold cross-validation (CV) accuracy in the training set was 90.4%, and the classification accuracies in ACRG-STAD and GSE84437 were 80.3% and 81.3%, respectively (Fig. 10). The weighted sensitivity, specificity, and F1 scores were more than 90% in the training set (tenfold CV) and exceeded 80% in both test sets (Fig. 10). These results reflect the reproducibility and predictability of the pathway-based GC classification method.

### Associations of pathway-based prognostic scores with survival prognosis and immunotherapy response

**Fig. 11:**
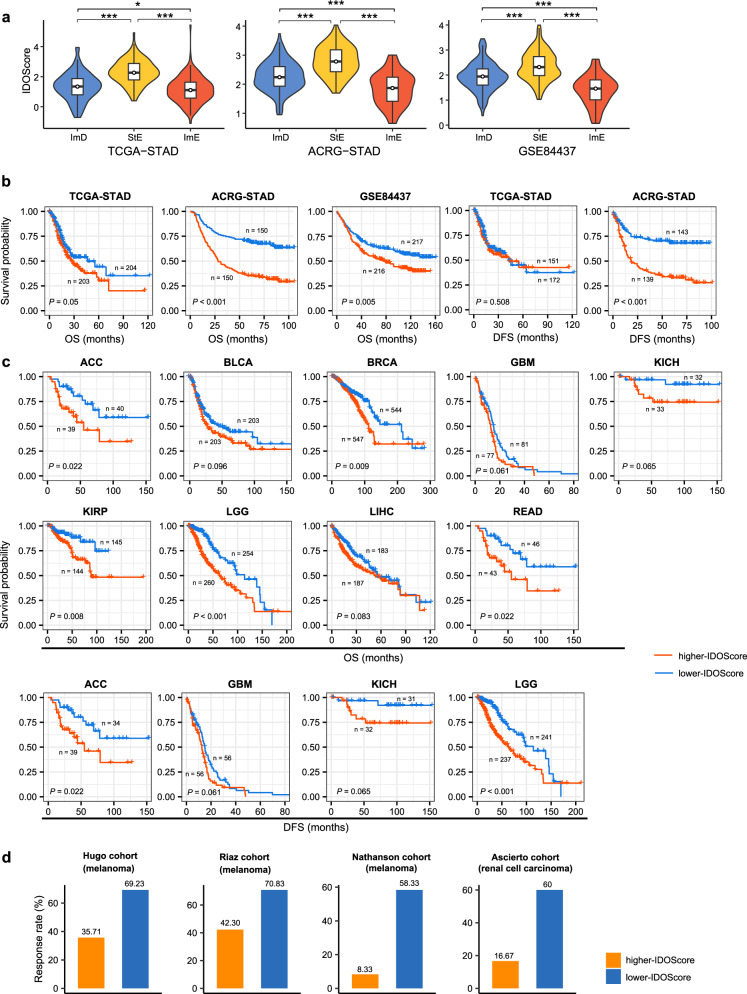
The prognostic model (IDOScore) developed based on the expression levels of four genes (*TAP2*, *SERPINB5*, *LTBP1*, and *LAMC1*) involved in immune, DNA damage repair, oncogenic, and stromal pathways. **a** Comparisons of the IDOScore values between the three GC subtypes. The one-tailed Mann–Whitney *U* test *P* values are indicated. Kaplan–Meier curves showing that the IDOScore is inversely correlated with survival prognosis in GC (**b**) and nine other cancer cohorts in TCGA (**c**) (log-rank test, *P* < 0.1). **d** Lower-IDOScore (< median) cancers showing significantly higher response rates than higher-IDOScore (>median) cancers in four cancer cohorts receiving immune checkpoint inhibitor treatment. ACC adrenocortical carcinoma, BLCA bladder urothelial carcinoma, BRCA breast invasive carcinoma, GBM glioblastoma multiforme, KICH kidney chromophobe, KIRP kidney renal papillary cell carcinoma, LGG brain lower grade glioma, LIHC liver hepatocellular carcinoma, READ rectum adenocarcinoma. *P < 0.05, **P < 0.01, ***P < 0.001.

To further prove the relatedness of the four types of pathways’ activities with survival prognosis and immunotherapy response, we developed a linear model (IDOScore) to assess the prognostic risk of GCs based on the expression levels of four genes, including *TAP2*, *SERPINB5*, *LTBP1*, and *LAMC1*. The four genes were involved in the four types of pathways based on which we clustered GCs, namely immune (*TAP2* in antigen processing and presentation), DNA damage repair (*SERPINB5* in p53 signaling), oncogenic (*LTBP1* in TGF-β signaling), and stromal (*LAMC1* in ECM–receptor interaction) pathways. The IDOScore was determined to be an adverse prognostic factor in terms of the positive association of immune and DNA damage repair signatures with prognosis and the negative association of oncogenic and stromal signatures with prognosis. Indeed, the IDOScore was the highest in StE and the lowest in ImE (*P* < 0.015) (Fig. 11a) and was inversely correlated with OS in all three GC cohorts (log-rank test, *P* ≤ 0.05) and with DFS in ACRG-STAD (*P* < 0.001) (Fig. 11b). Furthermore, we examined the association between the IDOScore and survival prognosis in other 29 TCGA cancer cohorts. Interestingly, the IDOScore was inversely associated with OS in 9 of the 29 cancer cohorts (ACC, BLCA, BRCA, GBM, KICH, KIRP, LGG, LIHC, and READ) and with DFS in four cancer cohorts (ACC, GBM, KICH, and LGG) (*P* < 0.1) (Fig. 11c). In addition, we investigated the association between the IDOScore and the response to ICIs in four cancer cohorts, namely the Hugo cohort (melanoma)^84^, Riaz cohort (melanoma)^85^, Nathanson cohort (melanoma)^86^, and Ascierto cohort (renal cell carcinoma)^87^. We found that lower-IDOScore (<median) cancers displayed significantly higher response rates than higher-IDOScore (>median) cancers in these cohorts (69.23% vs. 35.71% in the Hugo cohort, 70.83% vs. 42.30% in the Riaz cohort, 58.33% vs. 8.33% in the Nathanson cohort, and 60% vs. 16.67% in the Ascierto cohort) (Fig. 11d). These results are justified because the immune and DNA damage repair signatures are likely to have a positive association with PD-1/PD-L1/CTLA-4-directed immunotherapy^11,13^.

## Discussion

**Fig. 12:**
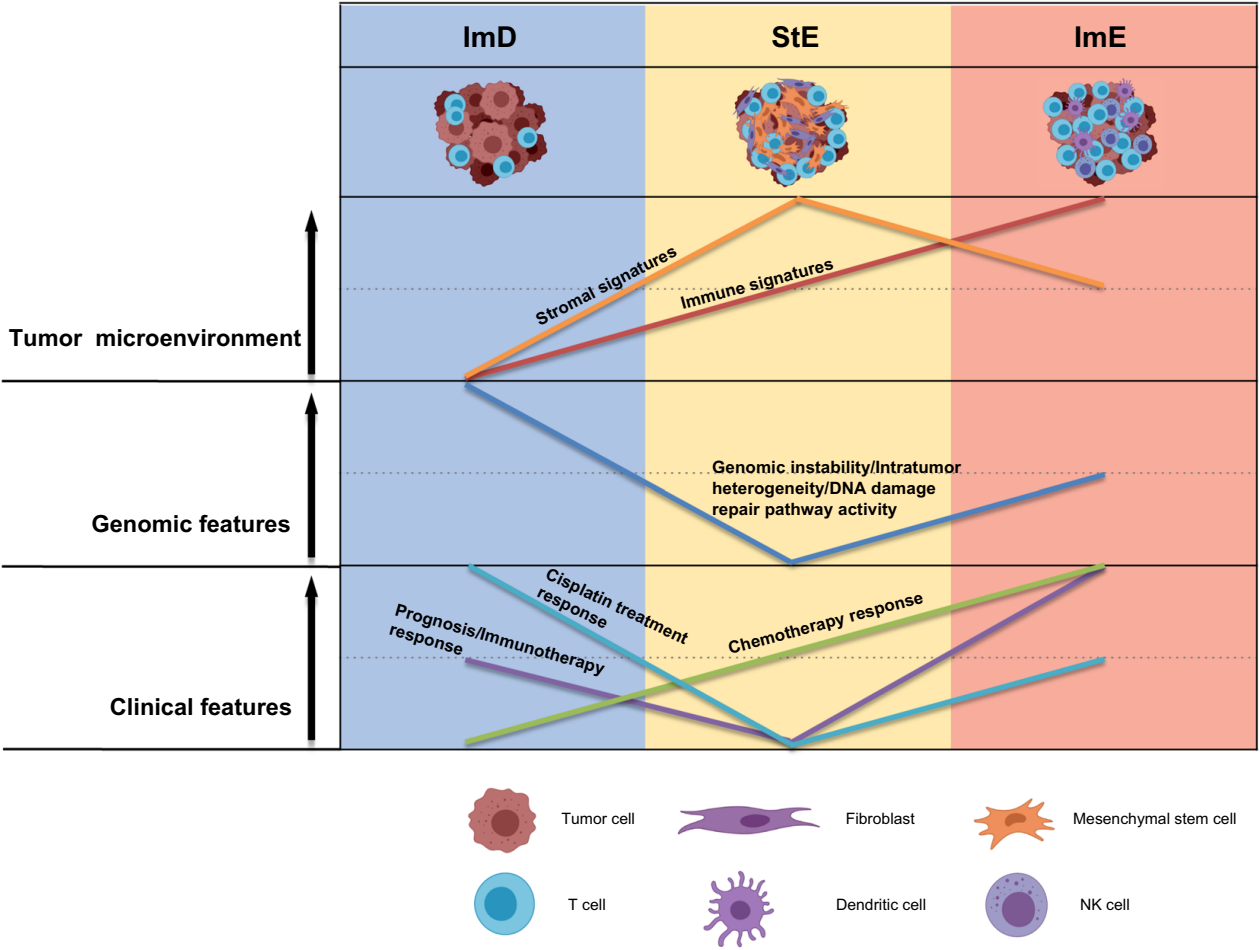
A summary of molecular and clinical features of the three GC subtypes. The three GC subtypes display significantly different molecular and clinical features. The figure was created with BioRender.com.

For the first time, we proposed a pathway-based classification method for GC. We classified GCs based on the enrichment levels of 15 pathways associated with immune, DNA damage repair, oncogenic, and stromal signatures. We identified three GC subtypes, namely ImD, StE, and ImE, and demonstrated that this classification method was stable and reproducible by testing it in three different datasets. ImD was characterized by low immune infiltration, high DNA damage repair activity, large-scale GI, high ITH, and frequent *TP53* mutations; StE was characterized by high stromal signatures, low DNA damage repair activity, high genomic stability, low ITH, strong oncogenic signatures, inferior response to ICIs, and poor prognosis; ImE was characterized by high immune infiltration, high DNA damage repair activity, small-scale GI, the prevalence of MSI, frequent *ARID1A* mutations, active response to ICIs, and favorable prognosis (Fig. 12). It is interesting to observe that both ImD and ImE have high TMB but significantly different immune infiltration. The main reason could be that different from ImE, ImD has frequent SCNAs that suppress antitumor immune responses^88^. This observation could explain why ImE responds better to ICIs than ImD since high immune infiltration indicates a more active response to immunotherapy^13^. The equal level of TMB but significantly different immunotherapy responses between ImE and ImD indicate that TMB is not necessarily a perfect determinant for predicting the response to ICIs. Another interesting observation was that although StE displayed higher levels of anti-tumor immune signatures than ImD, it had the worst prognosis among the three subtypes. The reason behind this could be the strongest stromal signatures presented in this subtype, which are associated with tumor invasion, metastasis, and drug resistance^62,63,89^. Also, although StE had stronger immune signatures than ImD, it had the worst immunotherapy response among the three subtypes. The main reason could be that stromal signatures, such as the TGF-β pathway^90^, can promote immune evasion in the tumor. Indeed, our data showed that StE had significantly stronger immunosuppressive signatures and lower ratios of immune-stimulatory/immune-inhibitory signatures than ImE and ImD. The strongest stromal and immunosuppressive signatures in StE may explain why this subtype has the worst immunotherapy response and clinical outcomes. It also indicates that the ratio of immune-stimulatory/immune-inhibitory signatures could be a better biomarker for predicting immunotherapy response than sole immune-stimulatory or immune-inhibitory signatures.

PD-L1 expression^10^, DNA mismatch repair deficiency or MSI^11^, high TMB^12^, and immune infiltration^13^ are indicative of an active response to ICIs. It indicates that ImE is likely to have the best response to ICIs since it is most highlighted by these features among the three subtypes. Indeed, we have predicted that ImE responded best to ICIs by using the TIDE algorithm^68^. A previous study demonstrated that TMB and a T cell-inflamed gene expression profile (T-GEP) were independent predictors for the response to ICIs and that patients having higher levels of both biomarkers exhibited the highest rates of response to ICIs^91^. As expected, T-GEP scores, which were the ssGSEA scores of 18 T cell-inflamed genes^92^, were the highest in ImE (Supplementary Fig. 6). Previous analyses have shown that TMB was significantly higher in ImD and ImE than StE. Again, these data collectively indicate the greatest immunotherapeutic benefit in ImE.

Previous studies have identified molecular subtypes of GC, such as four subtypes identified by integration of multi-omics data in TCGA: EBV-associated, MSI, GS, and CIN^5^, four subtypes defined based on EMT, MSI, and *TP53* signatures in ACRG: MSS/EMT, MSI, MSS/p53+, and MSS/p53-^6^, three subtypes defined by the expression levels of 29 immune genes: immune-high, immune-intermediate, and immune-low^15^, and three subtypes defined by the expression profiles of tumor microenvironment cells^16^. Compared to these classification methods, our method exhibits certain advantages. First, because the pathway enrichment score-based clustering integrates the expression levels of a set of genes into a single value, it exhibits higher stability and robustness than gene expression profiles-based clustering. Second, we used 15 signatures of pathways belonging to four types of pathways (immune, stromal, DNA damage repair, and oncogenic pathways), for the identification of GC subtypes. Evidently, our signatures are more comprehensive than those used in most of the previous methods, such as EMT, MSI, and *TP53* signatures used in ACRG^6^, an immune signature used by Park et al.^15^, immune and stromal signatures used by Zeng et al.^16^. As a result, our method captures better the comprehensive heterogeneity of stromal and immune microenvironment, genome integrity, and oncogenic signatures in GC. Furthermore, although TCGA identified molecular subtypes of GCs based on a comprehensive analysis of distinct data types, including somatic mutations, SCNAs, CpG methylation, mRNA, miRNA, and protein expression, this method is difficult to be applied in clinical practice due to a large cost on generating these data. Third, we comprehensively characterized molecular and clinical features associated with the GC subtypes we identified, including tumor immune microenvironment, stromal signatures, DNA damage repair activity, genome integrity, ITH, somatic mutation and SCNA profiles, protein expression profiles, tumor progression, response to chemotherapy and immunotherapy, and clinical outcomes. Thus, our identification of new GC subtypes may provide novel insights into tumor biology and has potential clinical implications for the precise management of GCs. Furthermore, although TCGA^5^ and ACRG^6^ also comprehensively characterized molecular and clinical features associated with the GC subtypes they identified, both studies did not correlate the GC subtypes with response to treatments, such as chemotherapy and immunotherapy. However, immunotherapy represents a promising direction in GC therapies. In addition, a recent study defined two GC molecular subtypes, namely mesenchymal phenotype and epithelial phenotype, based on genomic and proteomic data^93^. The mesenchymal phenotype exhibited shared characteristics with our StE subtype, including high genomic stability, strong EMT signature, resistance to standard chemotherapy, and poor prognosis. The epithelial phenotype had certain common characteristics with our ImD and ImE subtypes, such as high GI, high DNA damage repair activity, and response to standard chemotherapy. However, unlike our classification method, that classification did not capture the intratumor heterogeneity within the epithelial phenotype, namely significantly different tumor immune microenvironment, somatic mutation and SCNA profiles, response to immunotherapy and chemotherapy, and clinical outcomes. Finally, the IDOScore defined based on the expression levels of merely four genes involved in the immune, DNA damage repair, oncogenic, and stromal pathways, respectively, displayed excellent prediction power for survival prognosis and immunotherapy response in diverse cancers. The simplicity and effectiveness of IDOScore warrant its potential value in clinical practice.

In conclusion, we performed a new classification of GCs based on the activities of 15 immune, DNA damage repair, oncogenic, and stromal pathways. We identified three stable GC subtypes, which were distinguished by tumor immune microenvironment, stromal signatures, DNA damage repair activity, genome integrity, ITH, somatic mutation and SCNA profiles, oncogenic signatures, response to chemotherapy, and immunotherapy, and clinical outcomes. The identification of new GC subtypes provides novel insights into tumor biology and has potential clinical implications for the management of GCs.

## Methods

### Datasets

We downloaded three gene expression profiling and clinical datasets for GC, including TCGA-STAD, ACRG-STAD (GSE62254), and GSE84437. The TCGA-STAD dataset was downloaded from the genomic data commons (GDC) data portal (https://portal.gdc.cancer.gov/), and the other datasets were downloaded from the NCBI gene expression omnibus (https://www.ncbi.nlm.nih.gov/geo/). From the GDC data portal, we also downloaded the somatic mutation (level 3 and “maf” files), SCNA (“SNP6” files), protein expression (level 3), and methylation profiling (HM450) datasets for TCGA-STAD and gene expression profiling (level 3 and RSEM normalized) and clinical datasets for other 29 cancer cohorts. Besides, we obtained gene expression or somatic mutation profiling and clinical data for five cancer cohorts treated with ICIs from their associated publications, including the Samstein (gastrointestinal cancer)^46^, Hugo (melanoma)^84^, Riaz (melanoma)^85^, Nathanson (melanoma)^86^, and Ascierto cohorts (renal cell carcinoma)^87^. The Samstein cohort is a pan-cancer, for which we analyzed its subset of gastrointestinal cancers, including esophagogastric and colorectal cancers. A description of these datasets is shown in Supplementary Table 4.

### Quantification of the enrichment levels of pathways, immune signatures, and biological processes

We quantified the enrichment level of a pathway or biological process in a tumor sample using the ssGSEA score^26^ based on the expression levels of its marker genes and the enrichment level of an immune signature as the mean expression level of its marker genes. These pathways, immune signatures, and biological processes and their marker genes are shown in Supplementary Tables 5 and 6.

### Clustering

We clustered GCs based on the enrichment levels of 15 pathways using the consensus clustering algorithm^30^ in three GC datasets (TCGA-STAD, ACRG-STAD, and GSE84437). The 15 pathways were associated with the immune (natural killer cell-mediated cytotoxicity, antigen processing and presentation, T cell receptor signaling, B cell receptor signaling, and Fc gamma R-mediated phagocytosis), stromal (ECM-receptor interaction, focal adhesion, and tight junction), DNA damage repair (p53 signaling, mismatch repair, and homologous recombination), and oncogenic signatures (PI3K-Akt signaling, Wnt signaling, TGF-β signaling, and cell cycle). Consensus clustering^30^ evaluates the number of possible clusters and their members within a dataset. It implements subsampling from a set of samples and determines specified cluster counts (k). Next, it calculates the pairwise consensus values and stores them in a symmetrical consensus matrix for each k. This method has been frequently used for analyzing gene expression data^94^. We performed the clustering analyses using the R package “ConsensusClusterPlus” with the parameters: clusterAlg = “pam”, distance = “euclidean”, reps = 1000, pItem = 0.8, and pFeature = 1.

### Evaluation of immune score, stromal score, tumor purity, TMB, TAL, ITH, and SCNA

We used ESTIMATE^31^ to assess the immune score, stromal score, and tumor purity for each tumor sample. The immune score, stromal score, and tumor purity represent the immune infiltration level, stromal content, and proportion of tumor cells in the tumor bulk. TMB is the total count of non-synonymous somatic mutations in the tumor, and TAL is the tumor aneuploidy level evaluated by ABSOLUTE^95^. We used the MATH^96^ and DEPTH^97^ algorithms to evaluate ITH at the DNA and mRNA levels, respectively. The MATH ITH scores were calculated by using the function “math.score”^96^ in the R package “maftools” with the input of “maf” files, and the DEPTH ITH scores were calculated by using the R package “DEPTH” with the input of gene expression profiles in tumor and normal tissues. GISTIC2^98^ was utilized to calculate arm- and focal-level SCNAs and G-scores with the input of “SNP6” files.

### Gene ontology analysis

We used WGCNA^69^ to identify the gene modules (gene ontology) differentially enriched in GC subtypes with the input of gene expression profiles, sample classification, and survival (OS and DFS) status in tumors.

### Class prediction

We applied the XGBoost algorithm^83^ to predict the three GC subtypes by using TCGA-STAD as the training set and ACRG-STAD and GSE84437 as test sets. The classification accuracy and weighted sensitivity, specificity, and F1-score were reported. We performed the class prediction using the R package “xgboost” with the parameters: booster = “gbtree,” max_depth = 6, subsample = 0.8, objective = “multi:softmax,” and num_class = 3.

### The IDOScore model for assessing the prognostic risk of tumors

In ACRG-STAD, based on the enrichment levels (ssGSEA scores) of the 15 pathways used for clustering analyses, we selected five pathways in the Cox proportional hazards model by Lasso. The five pathways included natural killer cell-mediated cytotoxicity, antigen processing and presentation, TGF-β signaling, p53 signaling, and ECM-receptor interaction. For each of the five pathways, we identified 20 genes whose expression levels had the highest Spearman’s correlations with the pathway’s ssGSEA scores. From the 100 genes, we selected seven genes in the Cox proportional hazards model by using Lasso. The univariate Cox regression analysis showed that the expression of all the seven genes was significantly correlated with OS (*P* < 0.05). Finally, we selected four of the seven genes by the multivariable Cox regression model with backward stepwise selection. The four genes included *TAP2*, *SERPINB5*, *LTBP1*, and *LAMC1*, which were involved in the immune, DNA damage repair, oncogenic, and stromal pathways, respectively. Using the four genes as predictors, we built the IDOScore prognostic model as follows: IDOScore = −0.497 × Exp (*TAP2*) − 0.166 × Exp (*SERPINB5*) + 0.154 × Exp (*LTBP1*) + 0.571 × Exp (*LAMC1*), where Exp (*X*) denotes the expression level of gene *X*. We used the “cv.glmnet” function in the R package “glmnet” for the variable selection by Lasso in the Cox proportional hazards model and the “coxph” function in the R package “survival” for the univariate and multivariable Cox regression analyses. For the backward stepwise selection, we used the “stepAIC” function in the R package “MASS”.

### Evaluation of proportions of immune cell subsets in the TME

We used CIBERSORT^33^ to assess the proportions of 22 human immune cell subsets. We implemented the CIBERSORT algorithm with 1000 permutations using a threshold of *P* < 0.05 as the criteria for the success in deconvolution of a sample.

### Survival analysis

We used Kaplan–Meier curves to show the survival (OS and DFS) time differences between different groups and the log-rank test to assess the significance of survival time differences.

### Logistic regression analysis

We used logistic regression with three predictors (StE, ImE, and tumor purity) to predict the immune score, stromal score, and EMT signature score (high (>median) vs. low (<median)), respectively. Three predictors were binary variables, where tumor purity equals to 1 (high) or 0 (low), StE equals to 1 (the sample belonging to StE) or 0 (otherwise), and ImE equals 1 (the sample belonging to ImE) or 0 (otherwise). In performing the logistic regression analyses, we used the R function “glm” to fit the binary model.

### Statistical analysis

We used Student’s *t* test (two-tailed) to compare two classes of normally distributed data, including gene expression levels, protein expression levels, and the ratios of immune-stimulatory over immune-inhibitory signatures. The ratios were then log2-transformed geometric mean expression levels of the marker genes of immune-stimulatory signatures over those of immune-inhibitory signatures. In comparisons of two classes of other data that were not normally distributed, we used Mann–Whitney *U* test (one-tailed). In comparisons of three classes of normal and not normally distributed data, we used the ANOVA and Kruskal–Wallis (K–W) test, respectively. We utilized Fisher’s exact test or Chi-square test to analyze contingency tables. The FDR evaluated by the Benjamini-Hochberg method^99^ was used to adjust for multiple tests.

### Reporting summary

Further information on research design is available in the Nature Research Reporting Summary linked to this article.

## Supplementary information

Supplementary Information

Reporting Summary

## Data Availability

The data associated with this study were presented in Supplementary Tables, and the algorithm tool (PP-GCS) for predicting GC subtypes can be found at the website: https://cpu-wangx-lab.shinyapps.io/ppgcs/.

## Code availability

We performed all computational and statistical analyses in the R programming environment (version 3.6.1, https://cran.r-project.org/).
