# Supplementary material for: Identification of gastric cancer subtypes based on pathway clustering
Source: NPJ Precis Oncol. 2021 Jun 2;5:46. doi: 10.1038/s41698-021-00186-z (PMC8172826; doi:10.1038/s41698-021-00186-z)
Supplement: Supplementary file 1 — Supplementary Information [file 41698_2021_186_MOESM1_ESM.pdf]

Supplementary Figures

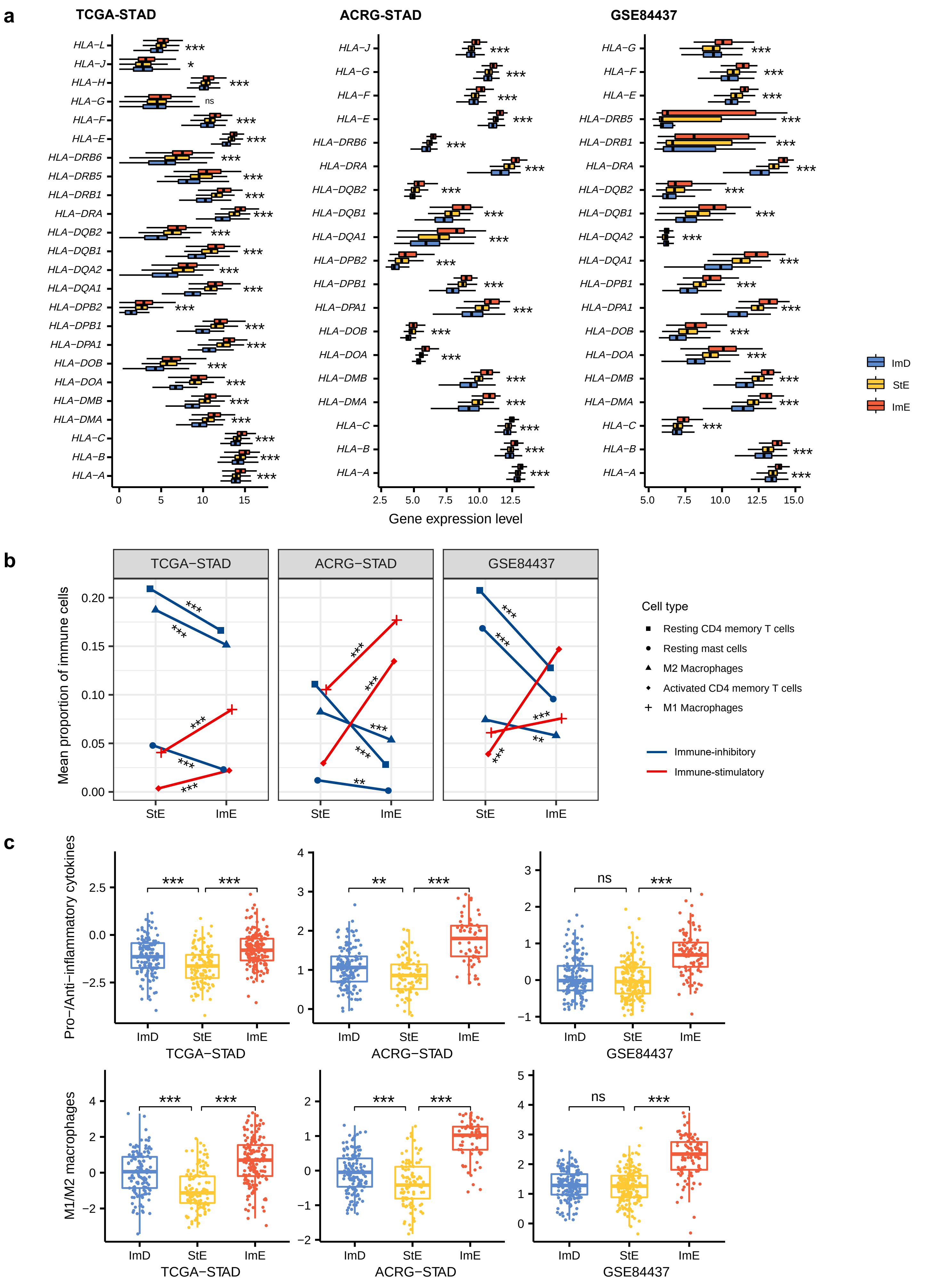

d

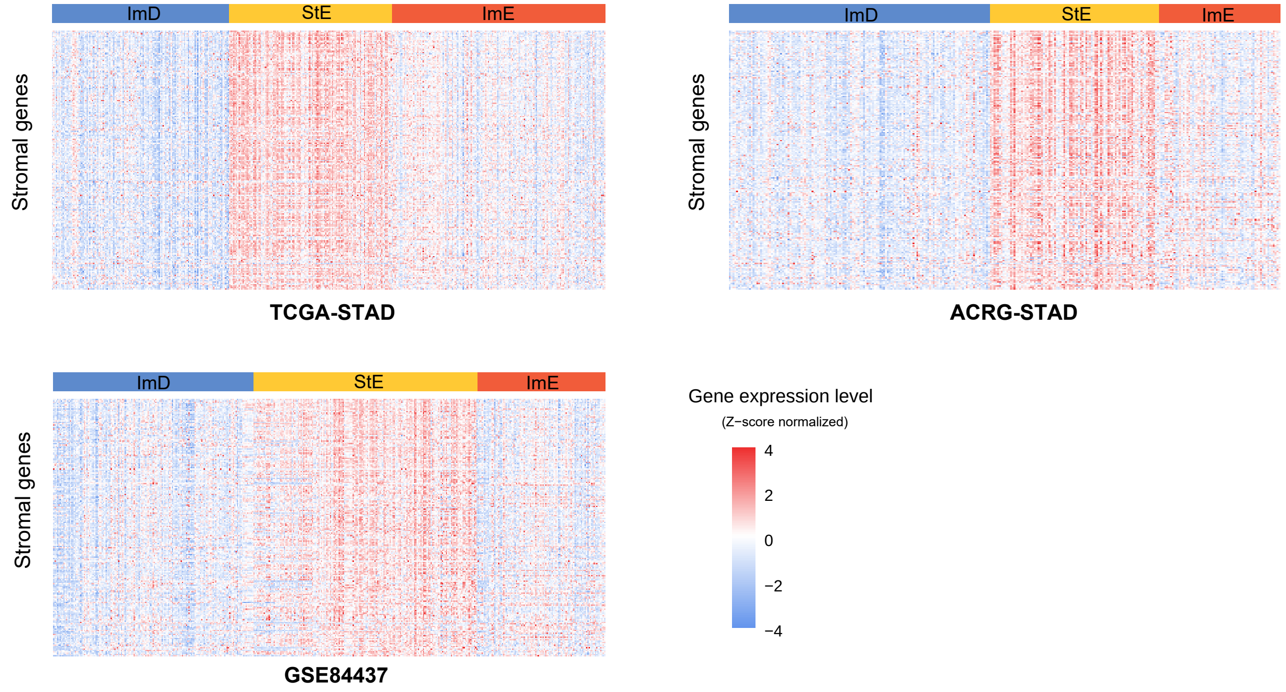

Supplementary Fig. 1. Comparisons of the expression levels of human leukocyte antigen (HLA) and stromal genes, immunosuppressive signatures, and ratios of immune-stimulatory to immune-inhibitory signatures between the three GC subtypes. (a) The expression levels of most HLA genes show the pattern: ImD < StE < ImE. The one-way ANOVA test *P* values are indicated. **(b)** The proportions of immunosuppressive cells (resting CD4 memory T cells, resting mast cells, and M2 macrophages) are significantly higher in StE than ImE, while the proportions of immune-stimulatory cells (activated CD4 memory T cells and M1 macrophages) are significantly lower in StE (one-tailed Mann–Whitney U test, $P < 0.01$). **(c)** The ratios of immune-stimulatory to immune-inhibitory signatures (pro-/anti-inflammatory cytokines and M1/M2 macrophages) are significantly lower in StE than ImE and ImD. The one-tailed Mann–Whitney U test *P* values are indicated. **(d)** Heatmap showing that most of the 194 stromal gene signatures are more highly expressed in StE than ImD and ImE (two-tailed student's t test, $P < 0.05$). * $P < 0.05$, ** $P < 0.01$, *** $P < 0.001$.

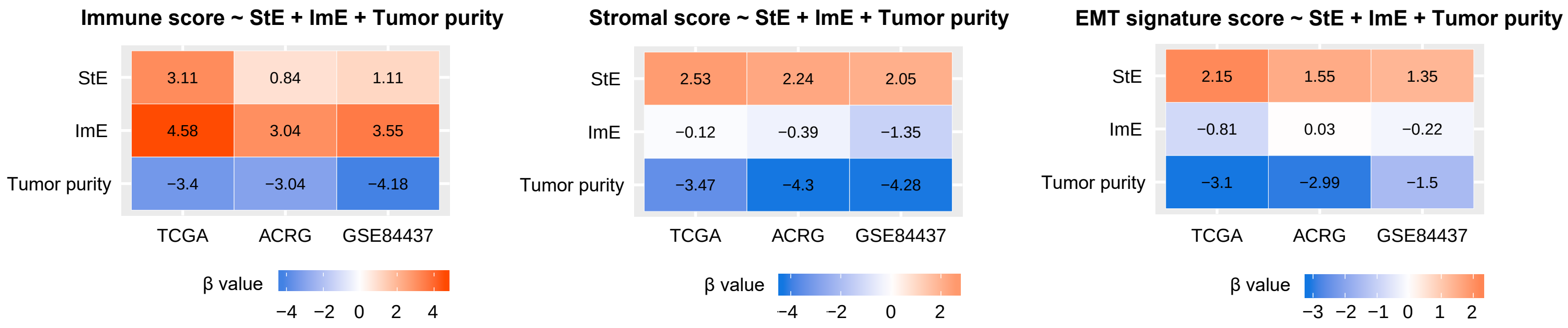

Supplementary Fig. 2. Logistic regression models with three predictors (StE, ImE, and tumor purity) to predict immune score, stromal score, and EMT signature score. The three predictors are binary variables, where tumor purity equals to 1 (high > median) or 0 (low < median), StE equals to 1 (the sample belonging to StE) or 0 (otherwise), and ImE equals to 1 (the sample belonging to ImE) or 0 (otherwise). The high (> median) versus low (< median) immune score, stromal score, and EMT signature score are predicted. The β values for the predictors are shown.

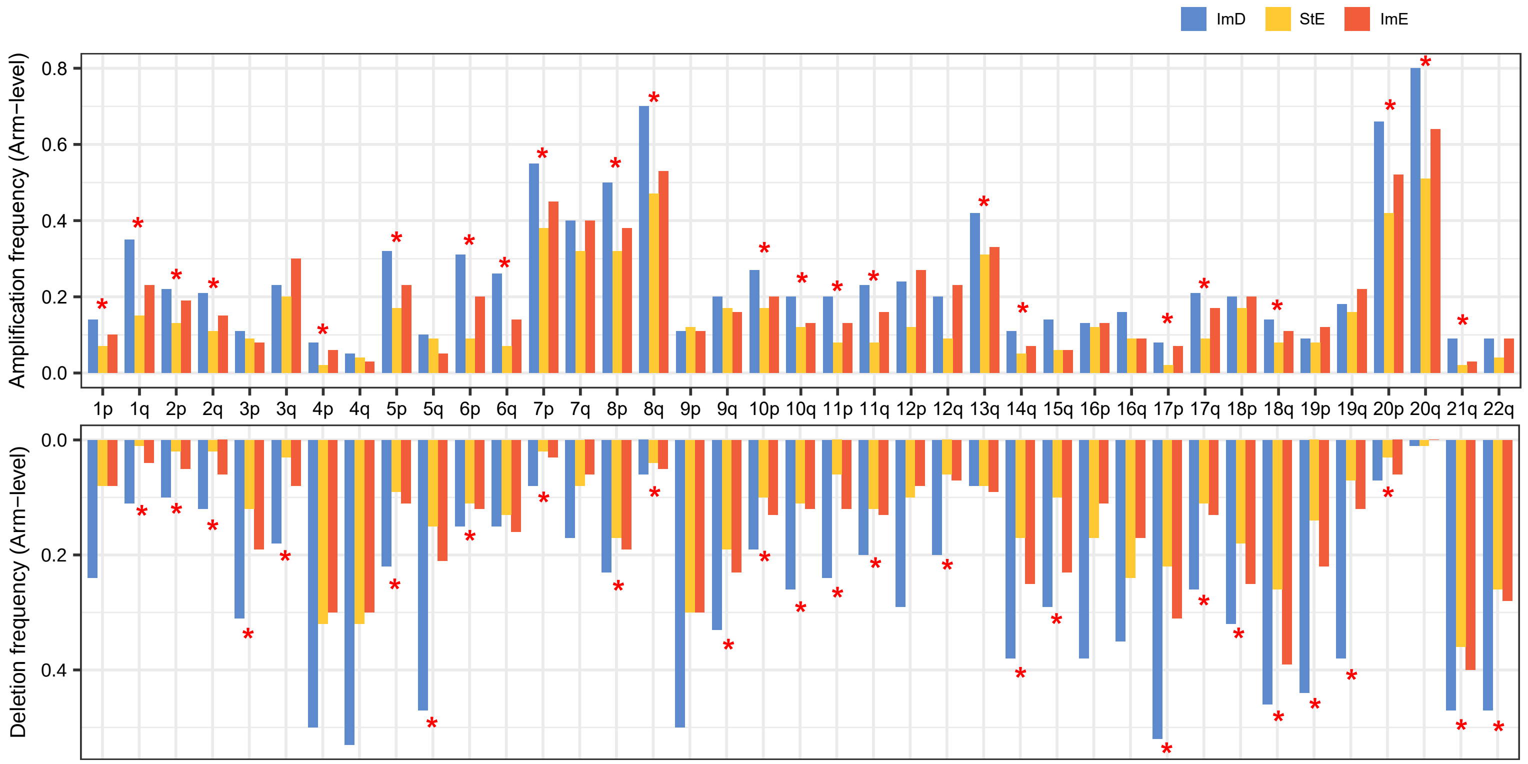

Supplementary Fig. 3. Comparison of the somatic copy number alteration (SCNA) levels between the three GC subtypes in TCGA-STAD. The red asterisks indicate the chromosome arms in which ImD and StE have the highest and lowest amplification or deletion frequencies among the three GC subtypes, respectively. The SCNA levels were calculated by GISTIC2 [1].

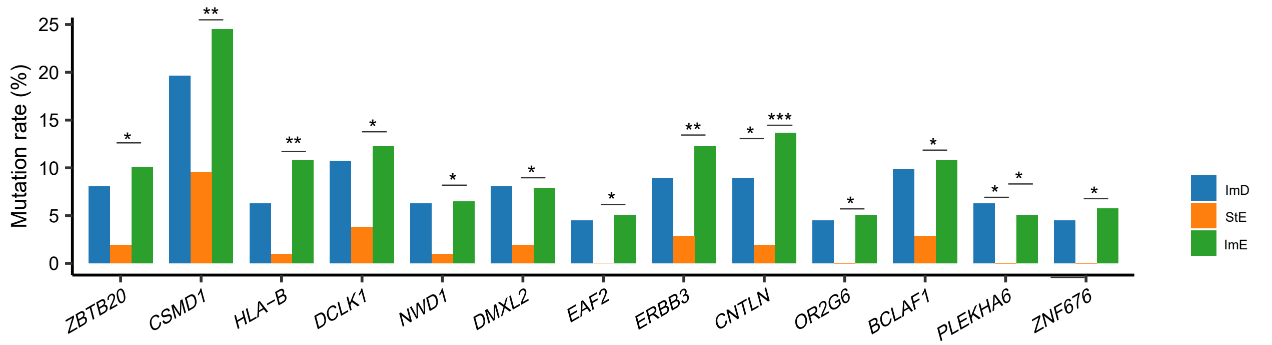

Supplementary Fig. 4. The genes showing higher mutation rates in ImE and/or ImD than StE (Fisher's exact test, *P* < 0.1, OR > 2). * *P* < 0.05, ** *P* < 0.01, *** *P* < 0.001.

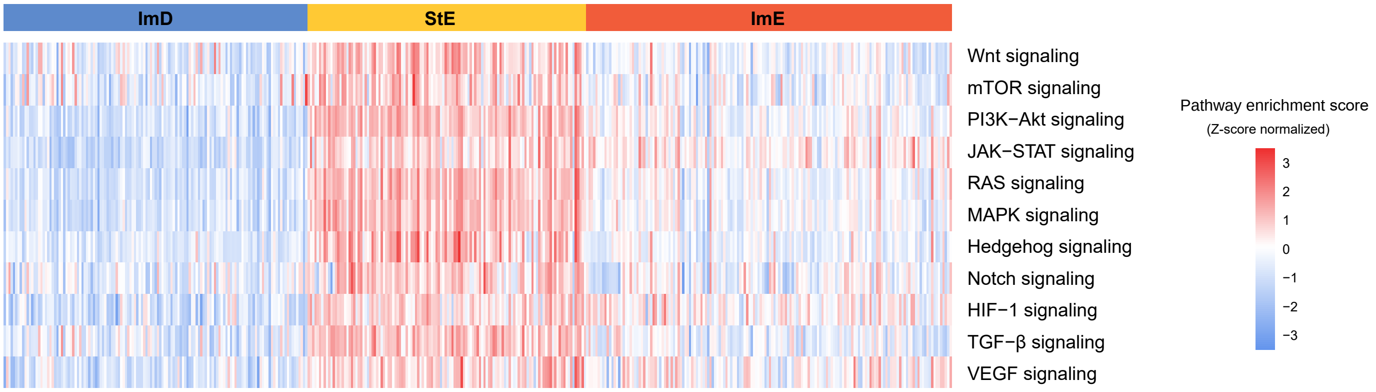

Supplementary Fig. 5. Heatmap showing the enrichment levels of 11 oncogenic pathways in the three GC subtypes in TCGA-STAD (one-tailed Mann–Whitney U test, *P* < 0.05).

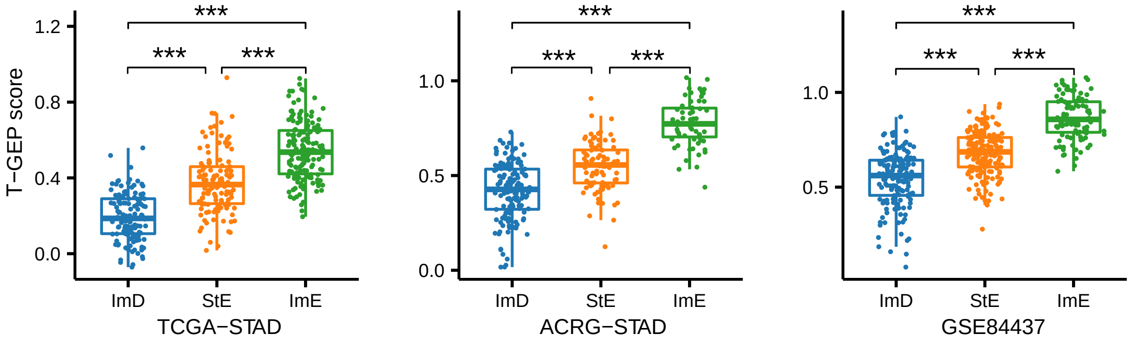

Supplementary Fig. 6. Comparisons of T cell-inflamed gene expression profile (T-GEP) scores between the three GC subtypes. One-tailed Mann–Whitney U test *P* values are indicated. * *P* < 0.05, ** *P* < 0.01, *** *P* < 0.001.

Supplementary Tables

Supplementary Table 1. Comparisons of mutation profiles of 172 driver genes between the three GC subtypes.

Gene	Mutation rate (%)			*P*-value (Fisher's exact test)
	ImD	StE	ImE	
PIK3CA	10.710	4.760	29.500	2.02E-07
TP53	64.290	33.330	44.600	1.98E-05
CR1	1.790	0.950	11.510	2.00E-04
CNTLN	8.930	1.900	13.670	0.003
HLA-B	6.250	0.950	10.790	0.005
ARID1A	16.960	14.290	30.220	0.005
CSMD1	19.640	9.520	24.460	0.009
KRAS	11.610	1.900	6.470	0.014
CIC	5.360	2.860	12.230	0.015
CASP8	1.790	1.900	8.630	0.015
XYLT2	6.250	NA	2.160	0.015
ZNF48	3.570	NA	6.470	0.016
PLEKHA6	6.250	NA	5.040	0.019
RHOA	0.890	8.570	3.600	0.021
B2M	5.360	0.950	8.630	0.024
AQP8	2.680	NA	5.760	0.024
ERBB3	8.930	2.860	12.230	0.025
RBM6	3.570	0.950	7.910	0.026
ZBTB20	8.040	1.900	10.070	0.027
ZNF676	4.460	NA	5.760	0.029
RPL10L	5.360	NA	5.040	0.031
KLC2	1.790	NA	5.040	0.037
PTEN	3.570	4.760	11.510	0.038
P2RY4	NA	NA	2.880	0.039
HLA-A	2.680	0.950	7.190	0.040
P4HTM	0.890	NA	4.320	0.040
KCTD9	0.890	NA	4.320	0.040
EAF2	4.460	NA	5.040	0.042
OR2G6	4.460	NA	5.040	0.042
GPSM3	5.360	NA	2.880	0.042
BCLAF1	9.820	2.860	10.790	0.045
RNF43	6.250	1.900	9.350	0.047
OR52E8	3.570	NA	5.040	0.048
DCLK1	10.710	3.810	12.230	0.050
MVK	7.140	0.950	3.600	0.054
RGS9BP	2.680	NA	NA	0.056
LRRN2	0.890	2.860	6.470	0.062
NWD1	6.250	0.950	6.470	0.063
CRYGA	4.460	NA	1.440	0.066

OR10S1	2.680	5.710	0.720	0.069
DMXL2	8.040	1.900	7.910	0.075
EPHA6	6.250	2.860	10.070	0.076
ZWINT	0.890	1.900	5.760	0.089
GYLTL1B	4.460	0.950	6.470	0.095
TBX4	0.890	NA	3.600	0.097
ZBTB7C	0.890	0.950	5.040	0.098
MICALCL	0.890	0.950	5.040	0.098
CDH1	4.460	12.380	9.350	0.099
FBXW7	8.930	2.860	9.350	0.099
CDKN2A	9.820	2.860	5.040	0.100
NLK	1.790	0.950	5.760	0.103
WNT16	2.680	NA	4.320	0.107
ZNF208	7.140	2.860	9.350	0.109
FGF13	6.250	0.950	4.320	0.120
FAM19A2	3.570	NA	3.600	0.129
MFRP	3.570	NA	3.600	0.129
CNBD1	7.140	2.860	2.160	0.145
ZFP36L2	3.570	NA	2.880	0.148
SPATA20	3.570	NA	2.880	0.148
ZNF878	3.570	NA	2.880	0.148
PGM5	5.360	3.810	10.070	0.149
MAP2K1	NA	0.950	2.880	0.158
OR4P4	5.360	0.950	2.160	0.161
FRMD4A	5.360	0.950	2.880	0.165
EDNRB	9.820	3.810	9.350	0.168
BRD8	2.680	0.950	5.040	0.181
PAX2	2.680	0.950	5.040	0.181
FHOD3	8.040	2.860	7.910	0.182
MNDA	5.360	0.950	4.320	0.191
RHOQ	0.890	NA	2.880	0.194
CCDC153	0.890	NA	2.880	0.194
SERPINI1	0.890	0.950	4.320	0.200
IRF2	5.360	1.900	6.470	0.214
IPO5	4.460	0.950	5.040	0.215
CDH9	8.040	3.810	9.350	0.219
AMPH	8.040	2.860	4.320	0.220
CD1E	4.460	0.950	1.440	0.223
OR5M3	6.250	1.900	5.760	0.226
CDH11	10.710	4.760	9.350	0.241
KLF3	5.360	2.860	7.910	0.245

TRIML2	6.250	2.860	2.160	0.247
OR4C16	4.460	0.950	2.880	0.261
DAO	4.460	0.950	2.880	0.261
TRAM1L1	4.460	0.950	3.600	0.276
ZNF804B	9.820	5.710	11.510	0.279
C4orf6	2.680	NA	1.440	0.280
LMAN1	2.680	NA	1.440	0.280
DDX59	2.680	NA	1.440	0.280
TAS2R10	2.680	NA	1.440	0.280
PNMT	1.790	NA	2.880	0.292
RAB4B	NA	0.950	NA	0.295
LARP7	4.460	0.950	2.160	0.298
TLR4	7.140	4.760	10.070	0.303
DDX17	0.890	0.950	3.600	0.308
TRIP4	0.890	0.950	3.600	0.308
ATP6V1B1	2.680	0.950	4.320	0.315
WASF3	2.680	NA	2.160	0.328
CETN3	2.680	NA	2.160	0.328
PAX6	3.570	1.900	5.760	0.337
RPS6KA6	4.460	1.900	5.760	0.339
CADM1	2.680	3.810	6.470	0.340
ELF3	0.890	3.810	3.600	0.342
SYCP1	5.360	1.900	2.880	0.346
FSHR	4.460	3.810	7.910	0.367
LHCGR	3.570	0.950	4.320	0.368
GNG12	1.790	NA	2.160	0.390
GAB2	1.790	NA	2.160	0.390
C7orf50	1.790	NA	2.160	0.390
FAM109B	1.790	NA	2.160	0.390
ADAMDEC1	0.890	NA	2.160	0.391
WDR49	8.040	3.810	7.190	0.400
KRT75	1.790	0.950	3.600	0.402
LRP1B	32.140	23.810	28.060	0.405
HDLBP	7.140	4.760	9.350	0.424
OR5D14	3.570	3.810	1.440	0.475
MCM8	3.570	0.950	2.880	0.485
ZNF124	3.570	0.950	2.880	0.485
FAM133A	3.570	0.950	2.160	0.498
C9orf131	3.570	0.950	2.160	0.498
HIATL1	1.790	NA	0.720	0.502
PROK2	1.790	NA	0.720	0.502

TMC4	1.790	NA	0.720	0.502
SMAD4	5.360	6.670	9.350	0.506
LARP4B	3.570	1.900	5.040	0.525
MYLK3	2.680	0.950	0.720	0.525
SLITRK6	7.140	4.760	8.630	0.547
ZNF626	4.460	1.900	2.160	0.554
HOXD8	1.790	NA	1.440	0.562
RAB42	1.790	NA	1.440	0.562
DDC	1.790	NA	1.440	0.562
KCNMB2	4.460	2.860	2.160	0.597
CYLC2	3.570	1.900	1.440	0.606
OR51A7	3.570	1.900	1.440	0.606
C19orf70	0.890	NA	NA	0.610
DCDC1	2.680	2.860	5.040	0.633
TGFBR2	2.680	2.860	5.040	0.633
DOLPP1	NA	0.950	1.440	0.637
OR2T12	2.680	0.950	1.440	0.676
TMEM41A	2.680	0.950	1.440	0.676
ERBB4	12.500	11.430	15.110	0.695
HGF	4.460	4.760	2.880	0.702
TUSC3	6.250	6.670	4.320	0.707
GLT6D1	0.890	0.950	2.160	0.738
LEMD1	0.890	0.950	2.160	0.738
OR2T4	3.570	1.900	2.160	0.761
OR2J3	3.570	1.900	2.160	0.761
FAM98C	0.890	1.900	2.160	0.774
WNT1	0.890	NA	1.440	0.781
AMY2B	3.570	1.900	3.600	0.797
PRKAB1	1.790	1.900	2.880	0.821
LCE2B	1.790	0.950	0.720	0.829
SPG20	4.460	4.760	6.470	0.834
NAPEPLD	1.790	0.950	2.160	0.880
OR51B5	2.680	1.900	2.880	0.918
MAGEE2	3.570	2.860	2.160	0.920
CDK1	0.890	NA	0.720	1.000
ROPN1B	NA	NA	0.720	1.000
MAP2K7	5.360	4.760	5.040	1.000
CST1	0.890	0.950	1.440	1.000
DSTN	0.890	0.950	0.720	1.000
CDC5L	4.460	3.810	4.320	1.000
CYP7B1	4.460	4.760	5.040	1.000

P2RX1	1.790	1.900	1.440	1.000
PRAMEF14	NA	NA	0.720	1.000
RGS18	1.790	0.950	1.440	1.000
TRMT6	0.890	0.950	1.440	1.000
OR10AG1	2.680	2.860	2.880	1.000
CBWD1	NA	NA	NA	NA
PLA2G1B	NA	NA	NA	NA
KLRK1	NA	NA	NA	NA
SOHLH2	NA	NA	NA	NA
CBWD6	NA	NA	NA	NA

Supplementary Table 2. Comparisons of expression levels of 219 proteins between the three GC subtypes.

Protein	Mean expression level (Z-score normalized)			*P*-value (One-way ANOVA test)
	ImD	StE	ImE	
14-3-3_epsilon	0.209	0.192	-0.143	0.009
Caspase-7_cleavedD198	-0.342	-0.566	0.696	6.75E-30
Cyclin_B1	0.466	-0.738	0.259	3.11E-21
4E-BP1	0.447	-0.704	0.233	4.56E-19
ACVRL1	-0.396	0.692	-0.296	5.90E-18
Rictor	-0.300	0.686	-0.341	6.31E-18
Caveolin-1	-0.339	0.700	-0.274	1.45E-17
PCNA	0.188	-0.659	0.391	2.81E-17
FASN	0.543	-0.635	0.081	4.31E-17
c-Kit	-0.342	0.646	-0.311	7.51E-17
PR	-0.102	0.682	-0.392	1.61E-16
MYH11	-0.424	0.594	-0.231	1.08E-15
Collagen_VI	-0.506	0.529	-0.202	3.15E-15
FoxM1	0.641	-0.445	0.005	2.46E-14
PREX1	-0.531	-0.174	0.452	5.29E-14
MSH2	0.454	-0.599	0.111	8.09E-14
ACC1	0.500	-0.572	0.018	8.09E-14
PEA15	-0.502	0.566	-0.101	1.00E-13
TFRC	0.151	-0.618	0.335	2.76E-13
XRCC1	0.126	-0.590	0.354	9.17E-13
TAZ	-0.256	0.597	-0.245	7.69E-12
Bcl-2	-0.511	0.493	-0.019	7.85E-12
FOXO3a_pS318_S321	-0.159	0.615	-0.265	8.72E-12
Cyclin_D1	-0.437	0.525	-0.077	2.63E-11
4E-BP1_pS65	-0.143	0.518	-0.325	6.32E-11
Cyclin_E1	0.483	-0.430	-0.048	2.02E-10
PI3K-p85	-0.514	0.038	0.340	2.23E-10
Chk2	0.248	-0.540	0.207	4.10E-10
S6	0.357	-0.506	0.151	4.37E-10
CDK1_pY15	0.476	-0.408	0.002	5.40E-10
MSH6	0.331	-0.510	0.159	1.21E-09
ASNS	0.257	-0.496	0.227	2.19E-09
HSP70	-0.413	0.449	-0.103	3.69E-09
eIF4E	0.234	-0.503	0.202	1.19E-08
Bim	0.176	-0.524	0.193	1.53E-08
Syk	-0.112	-0.443	0.342	1.59E-08
Bax	-0.279	-0.333	0.343	1.74E-08
Claudin-7	0.257	-0.482	0.144	2.56E-08
Caspase-3	0.382	-0.431	0.101	2.71E-08

cIAP	0.349	-0.452	0.097	4.07E-08
ACC_pS79	0.454	-0.288	-0.173	7.53E-08
PKC-delta_pS664	-0.332	0.463	-0.062	9.23E-08
PRDX1	-0.194	-0.277	0.353	1.29E-07
DIRAS3	-0.135	0.464	-0.220	1.53E-07
YAP_pS127	-0.047	0.374	-0.342	2.13E-07
CD26	-0.063	0.493	-0.214	2.44E-07
Jak2	-0.128	-0.388	0.279	2.56E-07
Smad1	0.275	-0.421	0.146	3.37E-07
BRCA2	-0.176	0.435	-0.238	4.27E-07
VEGFR2	-0.053	0.434	-0.275	4.29E-07
PKC-alpha_pS657	-0.172	0.444	-0.212	5.16E-07
Rictor_pT1135	-0.042	0.455	-0.248	5.81E-07
ER-alpha	-0.202	0.405	-0.244	5.95E-07
Rab11	-0.367	0.376	-0.071	8.92E-07
Acetyl-a-Tubulin-Lys40	0.365	0.047	-0.310	9.73E-07
CD20	-0.428	0.306	0.038	1.10E-06
DUSP4	0.340	-0.370	0.020	1.24E-06
eEF2	-0.088	-0.359	0.318	1.66E-06
Bap1-c-4	0.429	-0.280	-0.001	2.23E-06
SF2	0.251	-0.381	0.193	2.32E-06
IGF1R_pY1135_Y1136	0.063	0.377	-0.292	2.50E-06
PKC-alpha	-0.283	0.384	-0.134	3.52E-06
Lck	-0.227	-0.208	0.334	3.82E-06
p90RSK	0.005	-0.370	0.294	4.51E-06
Transglutaminase	-0.396	0.129	0.231	6.69E-06
RBM15	0.343	-0.322	0.119	7.48E-06
ETS-1	-0.395	0.201	0.173	8.41E-06
Src	0.240	-0.415	0.059	1.12E-05
E-Cadherin	0.273	-0.370	0.081	1.46E-05
HER3	0.341	0.094	-0.254	1.49E-05
ADAR1	0.446	-0.151	-0.095	1.77E-05
Smad3	-0.336	0.294	0.072	2.29E-05
alpha-Catenin	0.313	-0.341	0.063	2.29E-05
IRS1	0.320	0.209	-0.237	3.13E-05
Ku80	0.365	-0.268	0.033	3.30E-05
JNK_pT183_pY185	-0.251	0.337	-0.141	3.90E-05
N-Cadherin	-0.383	0.234	0.024	3.93E-05
GAPDH	-0.044	-0.272	0.300	4.15E-05
HER2	0.401	-0.089	-0.158	4.40E-05
ERK2	-0.383	0.089	0.196	4.95E-05

p21	-0.310	0.318	-0.075	5.57E-05
MEK1	-0.387	-0.004	0.184	8.70E-05
AMPK_alpha	-0.174	0.376	-0.116	9.20E-05
A-Raf	0.139	-0.319	0.198	9.95E-05
mTOR_pS2448	-0.170	0.333	-0.195	1.04E-04
14-3-3_beta	-0.307	0.298	0.055	1.30E-04
Bcl2A1	-0.210	0.342	-0.115	1.52E-04
Cyclin_E2	0.091	-0.372	0.120	1.53E-04
AR	-0.296	0.273	-0.109	1.87E-04
Akt_pT308	-0.352	0.214	-0.037	2.44E-04
eIF4G	0.377	-0.128	-0.071	2.61E-04
Akt	-0.276	0.301	-0.061	2.69E-04
GSK3-alpha-beta	0.276	-0.291	0.031	2.76E-04
JNK2	-0.357	0.208	0.047	2.80E-04
TTF1	-0.271	0.291	-0.067	2.88E-04
14-3-3_zeta	-0.129	-0.236	0.267	3.12E-04
Myosin-IIa_pS1943	-0.074	-0.218	0.281	3.16E-04
Raptor	-0.252	0.266	-0.156	3.52E-04
YAP	-0.088	0.300	-0.215	3.73E-04
PEA15_pS116	-0.324	0.180	0.142	3.93E-04
Rb_pS807_S811	0.272	-0.271	0.016	6.04E-04
S6_pS235_S236	0.198	-0.321	0.075	7.53E-04
Tuberin_pT1462	-0.121	0.296	-0.181	7.92E-04
BRD4	0.113	-0.288	0.201	7.98E-04
B-Raf_pS445	0.247	-0.285	0.000	8.14E-04
HER2_pY1248	0.318	-0.018	-0.175	9.12E-04
P16INK4A;CDKN2A	0.313	-0.130	-0.137	0.001
c-Myc	0.074	0.340	-0.148	0.001
LKB1	-0.203	0.302	-0.002	0.002
4E-BP1_pT70	0.214	-0.244	0.154	0.002
Smac	0.234	-0.258	0.101	0.002
53BP1	0.315	-0.166	-0.039	0.002
p38_MAPK	-0.260	-0.093	0.207	0.002
EGFR_pY1068	0.249	0.042	-0.214	0.002
GSK3_pS9	-0.163	0.268	-0.146	0.002
NF2	-0.293	-0.003	0.184	0.002
Notch1	0.303	-0.036	-0.157	0.003
Shc_pY317	0.276	0.100	-0.170	0.003
MIG-6	0.361	0.022	-0.083	0.003
PDK1_pS241	-0.026	-0.231	0.212	0.004
ERCC5	0.092	-0.268	0.159	0.004

PKC-pan_BetaII_pS660	-0.284	0.182	0.044	0.004
Bad_pS112	0.108	0.230	-0.198	0.004
p62-LCK-ligand	0.208	-0.249	0.047	0.004
IRF-1	-0.235	0.172	0.140	0.005
ARID1A	0.287	-0.166	-0.025	0.005
Chk2_pT68	0.272	0.018	-0.170	0.005
A-Raf_pS299	-0.205	0.236	-0.084	0.005
c-Jun_pS73	0.320	0.107	-0.095	0.005
Chk1_pS345	-0.257	0.217	0.015	0.005
eEF2K	0.299	0.002	-0.131	0.005
Paxillin	-0.065	0.255	-0.171	0.005
PRAS40_pT246	0.303	0.038	-0.129	0.006
COG3	0.240	-0.204	-0.017	0.006
Annexin-1	-0.280	0.109	0.101	0.008
Stathmin	-0.173	0.247	-0.071	0.008
Mre11	0.077	0.245	-0.162	0.009
EGFR_pY1173	-0.009	0.270	-0.124	0.010
Beclin	-0.050	0.286	-0.086	0.010
Annexin_VII	-0.120	0.264	-0.085	0.010
Fibronectin	-0.165	0.182	-0.187	0.010
p90RSK_pT359_S363	0.239	0.053	-0.157	0.012
GSK3-alpha-beta_pS21_S9	-0.140	0.217	-0.120	0.013
4E-BP1_pT37_T46	0.238	-0.112	-0.118	0.014
CD49b	0.128	-0.234	0.113	0.015
p53	0.273	-0.024	-0.113	0.015
STAT3_pY705	-0.133	0.179	-0.185	0.015
c-Abl	-0.067	0.195	-0.186	0.019
Src_pY527	0.055	0.125	-0.216	0.021
STAT5-alpha	-0.213	0.123	0.108	0.022
IGFBP2	0.185	0.093	-0.159	0.025
p27_pT157	-0.157	0.202	-0.084	0.026
PARP1	0.178	-0.507	0.137	0.026
P-Cadherin	0.172	0.174	-0.128	0.027
XBP1	0.122	0.178	-0.156	0.028
SCD	0.297	-0.018	-0.046	0.028
Akt_pS473	-0.178	0.178	-0.079	0.029
NF-kB-p65_pS536	0.008	0.179	-0.158	0.040
HER3_pY1289	0.115	0.160	-0.147	0.043
CD31	-0.238	0.075	0.066	0.043
Src_pY416	-0.227	-0.047	0.112	0.046
SHP-2_pY542	0.143	0.114	-0.148	0.050

FOXO3a	0.114	0.145	-0.136	0.051
Dvl3	-0.007	0.175	-0.151	0.052
ERCC1	-0.224	0.081	0.061	0.055
Chk1	-0.248	-0.050	0.072	0.057
p70S6K	-0.052	-0.169	0.148	0.058
MAPK_pT202_Y204	-0.112	0.161	-0.128	0.058
EGFR	0.144	0.107	-0.141	0.061
NDRG1_pT346	0.177	-0.102	-0.104	0.063
YB-1	-0.104	0.174	-0.100	0.065
TSC1	0.163	-0.038	-0.144	0.067
INPP4B	-0.111	0.185	-0.085	0.067
PI3K-p110-alpha	0.168	0.116	-0.122	0.068
Smad4	0.257	0.030	-0.043	0.071
Bid	-0.206	0.107	-0.087	0.079
Heregulin	0.062	0.248	-0.060	0.081
Rab25	0.192	0.023	-0.091	0.104
TIGAR	0.045	-0.204	0.026	0.121
N-Ras	0.145	0.095	-0.111	0.121
beta-Catenin	0.161	-0.125	0.000	0.128
p70S6K_pT389	0.139	0.151	-0.080	0.141
Rad51	0.065	-0.099	0.143	0.183
YB-1_pS102	0.136	-0.078	-0.091	0.196
DJ-1	-0.139	0.060	0.097	0.196
MEK1_pS217_S221	0.039	0.146	-0.087	0.200
PAI-1	0.103	-0.138	-0.056	0.237
Rad50	-0.140	0.022	0.084	0.245
PDK1	-0.096	-0.103	0.092	0.263
Chk1_pS296;CHEK1	0.059	0.080	-0.110	0.296
mTOR	0.012	-0.172	0.015	0.299
JAB1	-0.039	-0.212	0.232	0.315
C-Raf_pS338	0.072	-0.004	-0.121	0.316
ER-alpha_pS118	0.088	-0.116	0.017	0.319
Snail	0.136	0.040	-0.065	0.339
S6_pS240_S244	0.042	-0.151	0.010	0.362
PDCD4	0.119	-0.082	0.003	0.370
C-Raf	-0.109	-0.035	0.072	0.391
p38_pT180_Y182	-0.101	0.070	0.013	0.467
EPPK1	0.048	-0.122	0.000	0.474
PTEN	-0.048	-0.026	0.101	0.482
PARP_cleaved	0.115	0.007	-0.037	0.525
c-Met	0.059	0.083	-0.057	0.535

c-Met_pY1235	0.073	-0.068	-0.045	0.559
ATM	-0.093	-0.017	0.044	0.590
AMPK_pT172	0.100	0.045	-0.035	0.595
B-Raf	0.040	-0.097	0.011	0.606
Caspase-8	0.120	0.015	-0.007	0.626
p27_pT198	0.048	-0.042	0.077	0.631
Bak	-0.036	0.063	-0.050	0.665
Tuberin	-0.001	-0.073	0.049	0.668
p27	-0.050	0.068	0.053	0.676
Bcl-xL	-0.032	-0.092	0.023	0.697
GAB2	-0.028	0.082	0.041	0.740
Rb	0.084	0.034	0.004	0.844
CDK1	-0.049	-0.069	0.000	0.858
GATA3	0.050	-0.007	0.052	0.892
G6PD	0.038	-0.019	0.020	0.911
SETD2	0.023	0.038	0.015	0.985

Supplementary Table 3. The 11 oncogenic pathways and their gene sets used in this study.

Pathway	Gene set
Wnt signaling	*PORCN, WNT1, WNT2, WNT2B, WNT3, WNT3A, WNT4, WNT5A, WNT5B, WNT6, WNT7A, WNT7B, WNT8A, WNT8B, WNT9A, WNT9B, WNT10B, WNT10A, WNT11, WNT16, CER1, NOTUM, WIF1, SERPINF1, SOST, DKK1, DKK2, DKK4, SFRP1, SFRP2, SFRP4, SFRP5, RSPO1, RSPO2, RSPO3, RSPO4, LGR4, LGR5, LGR6, RNF43, ZNRF3, FZD1, FZD7, FZD2, FZD3, FZD4, FZD5, FZD8, FZD6, FZD10, FZD9, LRP5, LRP6, BAMBI, CSNK1E, TPTEP2-CSNK1E, DVL3, DVL2, DVL1, FRAT1, FRAT2, CSNK2A1, CSNK2A2, CSNK2A3, CSNK2B, NKD1, NKD2, CXXC4, SENP2, GSK3B, CTNNB1, AXIN1, AXIN2, APC, APC2, CSNK1A1L, CSNK1A1, TCF7, TCF7L1, TCF7L2, LEF1, CTNNBIP1, CBY1, CHD8, SOX17, CTBP1, CTBP2, CTNND2, CREBBP, EP300, RUVBL1, SMAD4, SMAD3, MAP3K7, NLK, MYC, JUN, FOSL1, CCND1, CCND2, CCND3, CCN4, PPARD, MMP7, PSEN1, PRKACA, PRKACB, PRKACG, TP53, SIAH1, CACYBP, SKP1, TBL1X, TBL1Y, TBL1XR1, BTRC, FBXW11, CUL1, RBX1, GPC4, ROR1, ROR2, RYK, VANGL2, VANGL1, PRICKLE1, PRICKLE2, PRICKLE4, PRICKLE3, INVS, DAAM1, DAAM2, RHOA, ROCK2, RAC1, RAC2, RAC3, MAPK8, MAPK10, MAPK9, PLCB1, PLCB2, PLCB3, PLCB4, CAMK2A, CAMK2D, CAMK2B, CAMK2G, PPP3CA, PPP3CB, PPP3CC, PPP3R1, PPP3R2, PRKCA, PRKCB, PRKCG, NFATC1, NFATC2, NFATC3, NFATC4*
mTOR signaling	*SLC7A5, SLC3A2, SLC38A9, ATP6V1A, ATP6V1B1, ATP6V1B2, ATP6V1C2, ATP6V1C1, ATP6V1D, ATP6V1E2, ATP6V1E1, ATP6V1F, ATP6V1G1, ATP6V1G3, ATP6V1G2, ATP6V1H, LAMTOR1, LAMTOR2, LAMTOR3, LAMTOR4, LAMTOR5, FLCN, FNIP1, FNIP2, RRAGA, RRAGB, RRAGC, RRAGD, SESN2, CASTOR1, CASTOR2, MIOS, SEH1L, WDR24, WDR59, SEC13, DEPDC5, NPRL2, NPRL3, SKP2, RNF152, RPTOR, AKT1S1, MTOR, DEPTOR, MLST8, TELO2, TTI1, CLIP1, GRB10, LPIN1, LPIN3, LPIN2, ULK1, ULK2, EIF4EBP1, EIF4E, EIF4E2, EIF4E1B, RPS6KB1, RPS6KB2, EIF4B, RPS6, STRADA, STRADB, STK11, CAB39, CAB39L, PRKAA1, PRKAA2, TSC1, TSC2, TBC1D7, TBC1D7-LOC100130357, RHEB, DDIT4, WNT1, WNT2, WNT2B, WNT3, WNT3A, WNT4, WNT5A, WNT5B, WNT6, WNT7A, WNT7B, WNT8A, WNT8B, WNT9A, WNT9B, WNT10B, WNT10A, WNT11, WNT16, FZD1, FZD7, FZD2, FZD3, FZD4, FZD5, FZD8, FZD6, FZD10, FZD9, LRP5, LRP6, DVL3, DVL2, DVL1, GSK3B, TNF, TNFRSF1A, IKBKB, INS, IGF1, INSR, IGF1R, GRB2, SOS1, SOS2, HRAS, KRAS, NRAS, BRAF, RAF1, MAP2K1, MAP2K2, MAPK1, MAPK3, RPS6KA3, RPS6KA1, RPS6KA2, RPS6KA6, IRS1, PIK3R1, PIK3R2, PIK3R3, PIK3CA, PIK3CD, PIK3CB, PTEN, PDPK1, AKT1, AKT2, AKT3, CHUK, MAPKAP1, RICTOR, PRR5, RHOA, PRKCA, PRKCB, PRKCG, SGK1, SGK1*
PI3K-Akt signaling	*EGF, TGFA, EREG, AREG, FGF1, FGF2, FGF3, FGF4, FGF17, FGF6, FGF7, FGF8, FGF9, FGF10, FGF16, FGF5, FGF18, FGF20, FGF22, FGF19, FGF21, FGF23, NGF, BDNF, NTF3, NTF4, INS, IGF1, IGF2, PDGFA, PDGFB, PDGFC, PDGFD, CSF1, KITLG, FLT3LG, VEGFA, VEGFB, PGF, VEGFC, VEGFD, HGF, ANGPT1, ANGPT2, ANGPT4, EFNA1, EFNA2, EFNA3, EFNA4, EFNA5, EGFR, ERBB2, ERBB3, ERBB4, FGFR1, FGFR2, FGFR3, FGFR4, NGFR, NTRK1, NTRK2, INSR, IGF1R, PDGFRA, PDGFRB, CSF1R, KIT, FLT3, FLT1, FLT4, KDR, MET, TEK, EPHA2, GRB2, SOS1, SOS2, HRAS, KRAS, NRAS, RAF1, MAP2K1, MAP2K2, MAPK1, MAPK3, IRS1, TLR2, TLR4, RAC1, IGH, SYK, CD19, PIK3AP1, GH1, GH2, CSH1, CSH2, PRL, OSM, IL2, IL3, IL6, IL4, IL7, IFNA1, IFNA2, IFNA4, IFNA5, IFNA6, IFNA7, IFNA8, IFNA10, IFNA13, IFNA14, IFNA16, IFNA17, IFNA21, IFNB1, EPO, CSF3, GHR, PRLR, OSMR, IL2RA, IL2RB, IL2RG, IL3RA, IL6R, IL4R, IL7R, IFNAR1, IFNAR2, EPOR, CSF3R, JAK1, JAK2, JAK3, COL1A1, COL1A2, COL2A1, COL4A2, COL4A4, COL4A6, COL4A1, COL4A5, COL4A3, COL6A1, COL6A2, COL6A3, COL6A6, COL6A5, COL9A1, COL9A2, COL9A3, LAMA1, LAMA2, LAMA3, LAMA5, LAMA4, LAMB1, LAMB2, LAMB3, LAMB4, LAMC1, LAMC2, LAMC3, CHAD, RELN, THBS1, COMP, THBS2, THBS3, THBS4, FN1, SPP1, VTN, TNC, TNN, TNR, TNXB, VWF, IBSP, ITGA1, ITGA2, ITGA2B, ITGA3, ITGA4, ITGA5, ITGA6, ITGA7, ITGA8, ITGA9, ITGA10, ITGA11, ITGAV, ITGB1, ITGB3, ITGB4, ITGB5, ITGB6, ITGB7, ITGB8, PTK2, PIK3CA, PIK3CD, PIK3CB, PIK3R1, PIK3R2, PIK3R3, F2R, CHRM1, CHRM2, LPAR1, LPAR2, LPAR3, LPAR4, LPAR5, LPAR6, GNB1, GNB2, GNB3, GNB4, GNB5, GNG2, GNG3, GNG4, GNG5, GNG7, GNG8, GNG10, GNG11, GNG12, GNG13, GNGT1, GNGT2, PIK3CG, PIK3R5, PIK3R6, PDPK1, STK11, PRKAA1, PRKAA2, DDIT4, TSC1, TSC2, RHEB, MLST8, MTOR, RPTOR, EIF4EBP1, EIF4E, EIF4E2, EIF4E1B, RPS6KB1, RPS6KB2, EIF4B, RPS6, PRKCA, PKN1, PKN2, PKN3, SGK1, SGK2, SGK3, C8orf44-SGK3, AKT1, AKT2, AKT3, MAGI1, MAGI2, PTEN, THEM4, PPP2CA, PPP2CB, PPP2R1B, PPP2R1A, PPP2R2A, PPP2R2B, PPP2R2C, PPP2R2D, PPP2R3B, PPP2R3C, PPP2R3A, PPP2R5B, PPP2R5C, PPP2R5D, PPP2R5E, PPP2R5A, HSP90AA1, HSP90AB1, HSP90B1, CDC37, CRTC2, PHLPP1, PHLPP2, TCL1A, TCL1B, MTCP1, NOS3, BRCA1, GSK3B, GYS2, GYS1, PCK1, PCK2, G6PC, G6PC2, G6PC3, MYC, CCND1, CDKN1A, CDKN1B, CDK2, CDK4, CDK6, CCND2, CCND3, CCNE1, CCNE2, FOXO3, RBL2, FASLG, BCL2L11, YWHAZ, YWHAB, YWHAQ, YWHAE, YWHAH, YWHAG, BAD, BCL2L1, BCL2, CASP9, CREB1, ATF2, ATF4, CREB3, CREB3L1, CREB3L2, CREB3L3, CREB3L4, CREB5, ATF6B, MCL1, RXRA, NR4A1, IKBKG, CHUK, IKBKB, RELA, NFKB1, MYB, MDM2, TP53*

JAK-STAT signaling	*IL2, IL3, IL4, IL5, IL6, IL7, IL9, IL10, IL11, IL12A, IL12B, IL13, IL15, IL17D, IL19, IL20, IL21, IL22, IL23A, IL24, IFNA1, IFNA2, IFNA4, IFNA5, IFNA6, IFNA7, IFNA8, IFNA10, IFNA13, IFNA14, IFNA16, IFNA17, IFNA21, IFNB1, IFNG, IFNE, IFNK, IFNL1, IFNL2, IFNL3, IFNW1, OSM, LIF, TSLP, CTF1, CSF2, CNTF, CSF3, EPO, GH1, GH2, CSH1, CSH2, LEP, THPO, PRL, EGF, PDGFA, PDGFB, IL2RA, IL2RB, IL2RG, IL3RA, IL4R, IL5RA, IL6R, IL7R, IL9R, IL10RA, IL10RB, IL11RA, IL12RB1, IL12RB2, IL13RA1, IL13RA2, IL15RA, IL20RA, IL20RB, IL21R, IL22RA1, IL22RA2, IL23R, IL27RA, IL6ST, IFNAR1, IFNAR2, IFNGR1, IFNGR2, IFNLR1, OSMR, LIFR, CRLF2, CNTFR, CSF2RA, CSF2RB, CSF3R, EPOR, GHR, LEPR, MPL, PRLR, EGFR, PDGFRA, PDGFRB, JAK1, JAK2, JAK3, TYK2, STAT1, STAT2, STAT3, STAT4, STAT5A, STAT5B, STAT6, CISH, SOCS1, SOCS2, SOCS3, SOCS4, SOCS5, SOCS7, SOCS6, BCL2, MCL1, BCL2L1, PIM1, MYC, CCND1, CCND2, CCND3, CDKN1A, AOX1, GFAP, STAM2, STAM, PTPN2, PTPN6, IRF9, CREBBP, EP300, PIAS1, PIAS2, PIAS3, PIAS4, FHL1, PTPN11, GRB2, SOS1, SOS2, HRAS, RAF1, PIK3CA, PIK3CD, PIK3CB, PIK3R1, PIK3R2, PIK3R3, AKT1, AKT2, AKT3, MTOR*
RAS signaling	*EGF, TGFA, FGF1, FGF2, FGF3, FGF4, FGF17, FGF6, FGF7, FGF8, FGF9, FGF10, FGF16, FGF5, FGF18, FGF20, FGF22, FGF19, FGF21, FGF23, NGF, BDNF, NTF3, NTF4, INS, IGF1, IGF2, PDGFA, PDGFB, PDGFC, PDGFD, CSF1, KITLG, FLT3LG, VEGFA, VEGFB, PGF, VEGFC, VEGFD, HGF, ANGPT1, ANGPT2, ANGPT4, EFNA1, EFNA2, EFNA3, EFNA4, EFNA5, EGFR, FGFR1, FGFR2, FGFR3, FGFR4, NGFR, NTRK1, NTRK2, INSR, IGF1R, PDGFRA, PDGFRB, CSF1R, KIT, FLT3, FLT1, FLT4, KDR, MET, TEK, EPHA2, GRB2, GAB1, GAB2, SHC1, SHC2, SHC3, SHC4, PTPN11, SOS1, SOS2, PLCG1, PLCG2, RASGRP1, RASGRP2, RASGRP3, RASGRP4, ZAP70, LAT, HTR7, GNB1, GNB2, GNB3, GNB4, GNB5, GNG2, GNG3, GNG4, GNG5, GNG7, GNG8, GNG10, GNG11, GNG12, GNG13, GNGT1, GNGT2, PRKACA, PRKACB, PRKACG, RASGRF1, RASGRF2, GRIN1, GRIN2A, GRIN2B, CALML3, CALM2, CALM3, CALM1, CALML6, CALML5, CALML4, HRAS, KRAS, NRAS, MRAS, RRAS, RRAS2, NF1, RASA1, RASA2, RASA3, RASA4, RASA4B, SYNGAP1, RASAL1, RASAL2, RASAL3, RASSF1, RASSF5, STK4, TIAM1, RAC1, RAC2, RAC3, PAK1, PAK2, PAK3, PAK4, PAK5, PAK6, BUB1B-PAK6, RHOA, PIK3CA, PIK3CD, PIK3CB, PIK3R1, PIK3R2, PIK3R3, AKT1, AKT2, AKT3, IKBKG, CHUK, IKBKB, NFKB1, RELA, BAD, BCL2L1, FOXO4, FASLG, AFDN, SHOC2, RAF1, MAP2K1, MAP2K2, MAPK1, MAPK3, PLA1A, PLA2G10, PLA2G2D, PLA2G2E, PLA2G3, PLA2G2F, PLA2G12A, PLA2G12B, PLA2G1B, PLA2G5, PLA2G2A, PLA2G2C, PLA2G4E, PLA2G4A, JMJD7-PLA2G4B, PLA2G4B, PLA2G4C, PLA2G4D, PLA2G4F, PLA2G6, PLAAT3, ELK1, ETS1, ETS2, BRAP, KSR1, KSR2, RAPGEF5, RAP1A, RAP1B, RALGDS, RGL1, RGL2, RALA, RALB, MAPK8, MAPK10, MAPK9, EXOC2, TBK1, REL, PLD1, PLD2, RALBP1, CDC42, PLCE1, PRKCA, PRKCB, PRKCG, RIN1, ABL1, ABL2, RAB5A, RAB5B, RAB5C, ARF6*
MAPK signaling	*CACNA1A, CACNA1B, CACNA1C, CACNA1D, CACNA1E, CACNA1F, CACNA1G, CACNA1H, CACNA1I, CACNA1S, CACNA2D1, CACNA2D2, CACNA2D3, CACNA2D4, CACNB1, CACNB2, CACNB3, CACNB4, CACNG1, CACNG2, CACNG3, CACNG4, CACNG5, CACNG6, CACNG7, CACNG8, PRKACA, PRKACB, PRKACG, PRKCA, PRKCB, PRKCG, GNA12, GNG12, PPP3CA, PPP3CB, PPP3CC, PPP3R1, PPP3R2, RASGRF1, RASGRF2, RASGRP1, RASGRP2, RASGRP3, RASGRP4, RAPGEF2, NF1, RASA1, RASA2, RAP1A, RAP1B, EGF, TGFA, EREG, AREG, FGF1, FGF2, FGF3, FGF4, FGF17, FGF6, FGF7, FGF8, FGF9, FGF10, FGF16, FGF5, FGF18, FGF20, FGF22, FGF19, FGF21, FGF23, NGF, BDNF, NTF3, NTF4, INS, IGF1, IGF2, PDGFA, PDGFB, PDGFC, PDGFD, CSF1, KITLG, FLT3LG, VEGFA, VEGFB, PGF, VEGFC, VEGFD, HGF, ANGPT1, ANGPT2, ANGPT4, EFNA1, EFNA2, EFNA3, EFNA4, EFNA5, EGFR, ERBB2, ERBB3, ERBB4, FGFR1, FGFR2, FGFR3, FGFR4, NGFR, NTRK1, NTRK2, INSR, IGF1R, PDGFRA, PDGFRB, CSF1R, KIT, FLT3, FLT1, FLT4, KDR, MET, TEK, EPHA2, GRB2, SOS1, SOS2, HRAS, KRAS, NRAS, RRAS, RRAS2, MRAS, ARAF, BRAF, RAF1, MAP2K1, MAP2K2, LAMTOR3, MAPK1, MAPK3, MKNK1, MKNK2, RPS6KA3, RPS6KA1, RPS6KA2, RPS6KA6, ATF4, ELK1, ELK4, MYC, SRF, FOS, MAPT, STMN1, PLA2G4E, PLA2G4A, JMJD7-PLA2G4B, PLA2G4B, PLA2G4C, PLA2G4D, PLA2G4F, TNF, IL1A, IL1B, TGFB1, TGFB2, TGFB3, TNFRSF1A, IL1R1, IL1RAP, TGFBR1, TGFBR2, FASLG, FAS, CD14, RAC1, RAC2, RAC3, CDC42, TRADD, CASP3, TRAF2, DAXX, MYD88, IRAK1, IRAK4, TRAF6, GADD45A, GADD45B, GADD45G, TAB1, TAB2, ECSIT, MAP4K3, MAP4K4, MAP4K1, PAK1, PAK2, STK4, STK3, MAP4K2, MAP3K8, MAP3K1, MAP3K11, MAP3K2, MAP3K3, MAP3K13, MAP3K12, MAP3K20, MAP3K6, MAP3K5, MAP3K7, MAP3K4, TAOK2, TAOK3, TAOK1, MAP2K4, MAP2K7, MAP2K3, MAP2K6, MAPK8IP1, MAPK8IP2, MAPK8IP3, FLNA, FLNC, FLNB, CRK, CRKL, ARRB1, ARRB2, MAPK8, MAPK10, MAPK9, MAPK11, MAPK12, MAPK13, MAPK14, MAPKAPK5, MAPKAPK2, MAPKAPK3, RPS6KA5, RPS6KA4, CDC25B, NFATC1, NFATC3, JUN, JUND, ATF2, TP53, DDIT3, MAX, MEF2C, HSPB1, AKT1, AKT2, AKT3, PPM1A, PTPRR, PTPN5, PTPN7, DUSP1, DUSP4, DUSP2, DUSP7, DUSP8, DUSP5, DUSP16, DUSP6, DUSP9, DUSP10, DUSP3, PPP5C, PPM1B, HSPA8, HSPA1A, HSPA2, HSPA1L, HSPA1B, HSPA6, MECOM, MAP2K5, MAPK7, NR4A1, MAP3K14, CHUK, IKBKB, IKBKG, NLK, NFKB1, NFKB2, RELA, RELB*

Hedgehog signaling	*PTCH1, PTCH2, SMO, GPR161, PRKACA, PRKACB, PRKACG, CSNK1A1L, CSNK1A1, CSNK1G2, CSNK1G3, CSNK1G1, CSNK1D, CSNK1E, TPTEP2-CSNK1E, GSK3B, GLI1, GLI2, GLI3, SUFU, KIF7, HHIP, CCND1, CCND2, BCL2, CUL1, BTRC, FBXW11, HHAT, HHATL, SHH, IHH, DHH, DISP1, SCUBE2, BOC, CDON, GAS1, LRP2, MOSMO, MEGF8, MGRN1, SMURF1, SMURF2, GRK2, GRK3, EVC, EVC2, EFCAB7, IQCE, ARRB1, ARRB2, KIF3A, CUL3, SPOP, SPOPL*
Notch signaling	*DLL3, DLL1, DLL4, JAG1, JAG2, MFNG, LFNG, RFNG, NOTCH1, NOTCH2, NOTCH3, NOTCH4, RBPJL, RBPJ, HES1, HES5, HEYL, HEY1, HEY2, PTCRA, DVL3, DVL2, DVL1, NUMB, NUMBL, DTX2, DTX3L, DTX1, DTX3, DTX4, ADAM17, PSEN1, PSEN2, PSENEN, NCSTN, APH1A, APH1B, MAML3, MAML2, MAML1, CREBBP, EP300, KAT2B, KAT2A, SNW1, CTBP1, CTBP2, TLE7, TLE1, TLE2, TLE3, TLE4, TLE6, NCOR2, CIR1, HDAC1, HDAC2, ATXN1L, ATXN1*
HIF-1 signaling	*IL6, IL6R, STAT3, TLR4, IFNG, IFNGR1, IFNGR2, RELA, NFKB1, INS, EGF, IGF1, INSR, EGFR, IGF1R, ERBB2, MAP2K1, MAP2K2, MAPK1, MAPK3, MKNK1, MKNK2, PIK3CA, PIK3CD, PIK3CB, PIK3R1, PIK3R2, PIK3R3, AKT1, AKT2, AKT3, MTOR, EIF4EBP1, EIF4E, EIF4E2, EIF4E1B, RPS6KB1, RPS6KB2, RPS6, HIF1A, VHL, RBX1, ELOC, ELOB, CUL2, EGLN1, EGLN3, EGLN2, ARNT, CREBBP, EP300, CYBB, PLCG1, PLCG2, PRKCA, PRKCB, PRKCG, CAMK2A, CAMK2D, CAMK2B, CAMK2G, TIMP1, LTBR, EPO, TF, TFRC, VEGFA, FLT1, SERPINE1, ANGPT1, ANGPT2, ANGPT4, TEK, EDN1, NOS2, NOS3, HMOX1, NPPA, SLC2A1, PDK1, HK3, HK1, HK2, HKDC1, PFKM, PFKP, PFKL, GAPDH, ALDOC, ALDOA, ALDOB, ENO3, ENO2, ENO1, ENO4, PGK2, PGK1, PFKFB3, LDHAL6A, LDHAL6B, LDHA, LDHB, LDHC, BCL2, CDKN1A, CDKN1B, PDHA2, PDHA1, PDHB*
TGF-β signaling	*CHRD, NOG, NBL1, MICOS10-NBL1, GREM1, GREM2, THBS1, DCN, FMOD, LEFTY1, LEFTY2, FST, BMP2, BMP4, BMP6, INHBB, BMP5, BMP7, BMP8B, BMP8A, GDF5, GDF6, GDF7, AMH, THSD4, FBN1, LTBP1, TGFB1, TGFB2, TGFB3, INHBA, INHBC, INHBE, NODAL, NEO1, HJV, BMPR1A, BMPR1B, ACVR1, BMPR2, ACVR2A, RGMA, RGMB, AMHR2, TGFBR1, TGFBR2, ACVR1B, ACVR2B, ACVR1C, BAMBI, SMAD1, SMAD5, SMAD9, SMAD2, SMAD3, SMAD4, SMAD6, SMAD7, SMURF1, SMURF2, ZFYVE9, ZFYVE16, HAMP, ID1, ID2, ID3, ID4, RBL1, E2F4, E2F5, TFDP1, CREBBP, EP300, SP1, TGIF1, TGIF2, MYC, CDKN2B, PITX2, RBX1, CUL1, SKP1, MAPK1, MAPK3, IFNG, TNF, RHOA, ROCK1, PPP2R1B, PPP2R1A, PPP2CA, PPP2CB, RPS6KB1, RPS6KB2*
VEGF signaling	*VEGFA, KDR, SH2D2A, PLCG1, PLCG2, PRKCA, PRKCB, PRKCG, SPHK1, SPHK2, HRAS, KRAS, NRAS, RAF1, MAP2K1, MAP2K2, MAPK1, MAPK3, PLA2G4E, PLA2G4A, JMJD7-PLA2G4B, PLA2G4B, PLA2G4C, PLA2G4D, PLA2G4F, PPP3CA, PPP3CB, PPP3CC, PPP3R1, PPP3R2, NFATC2, PTGS2, PTK2, SHC2, PXN, CDC42, MAPK11, MAPK12, MAPK13, MAPK14, MAPKAPK2, MAPKAPK3, HSPB1, SRC, PIK3CA, PIK3CD, PIK3CB, PIK3R1, PIK3R2, PIK3R3, RAC1, RAC2, RAC3, AKT1, AKT2, AKT3, NOS3, CASP9, BAD*

Supplementary Table 4. A summary of the datasets used in this study.

Dataset	Cancer type	Sample size	Source
TCGA-STAD	stomach adenocarcinoma	415	TCGA
ACRG-STAD	stomach adenocarcinoma	300	GEO (GSE62254)
GSE84437	stomach adenocarcinoma	433	GEO (GSE84437)
ACC	adrenocortical carcinoma	79	TCGA
BLCA	bladder urothelial carcinoma	408	TCGA
BRCA	breast invasive carcinoma	1100	TCGA
CESC	cervical squamous cell carcinoma and endocervical adenocarcinoma	306	TCGA
CHOL	cholangiocarcinoma	36	TCGA
COAD	colon adenocarcinoma	287	TCGA
DLBC	lymphoid neoplasm diffuse large B-cell lymphoma	48	TCGA
ESCA	esophageal carcinoma	185	TCGA
GBM	glioblastoma multiforme	166	TCGA
HNSC	head and Neck squamous cell carcinoma	522	TCGA
KICH	kidney chromophobe	66	TCGA
KIRC	kidney renal clear cell carcinoma	534	TCGA
KIRP	kidney renal papillary cell carcinoma	291	TCGA
LAML	acute myeloid leukemia	173	TCGA
LGG	brain lower grade glioma	530	TCGA
LIHC	liver hepatocellular carcinoma	373	TCGA
LUAD	lung adenocarcinoma	517	TCGA
LUSC	lung squamous cell carcinoma	501	TCGA
OV	ovarian serous cystadenocarcinoma	307	TCGA
PAAD	pancreatic adenocarcinoma	179	TCGA
PRAD	prostate adenocarcinoma	498	TCGA
READ	rectum adenocarcinoma	95	TCGA
SARC	sarcoma	263	TCGA
SKCM	skin cutaneous melanoma	472	TCGA
TGCT	testicular germ cell tumors	156	TCGA
THCA	thyroid carcinoma	509	TCGA
THYM	thymoma	120	TCGA
UCEC	uterine corpus endometrial carcinoma	370	TCGA
UVM	uveal melanoma	80	TCGA
Samstein cohort	gastrointestinal adenocarcinoma	227	Ref. [1]
Hugo cohort	melanoma	28	GEO (GSE78220)
Riaz cohort	melanoma	58	GEO (GSE91061)
Nathanson cohort	melanoma	24	Ref. [2]
Ascierto cohort	renal cell carcinoma	11	GEO (GSE67501)

References

[1] Samstein, R.M., et al., Tumor mutational load predicts survival after immunotherapy across multiple cancer types. Nat Genet, 2019. 51(2): p. 202-206.

[2] Nathanson, T., et al., Somatic Mutations and Neoepitope Homology in Melanomas Treated with CTLA-4 Blockade. Cancer Immunol Res, 2017. 5(1): p. 84-91.

Supplementary Table 5. The 15 pathways and their gene sets used in this study.

Pathway	Gene set
Natural killer cell mediated cytotoxicity	*HLA-A, HLA-B, HLA-C, HLA-G, HLA-E, KIR3DL2, KIR3DL1, KIR3DL3, KIR2DL2, KIR2DL1, KIR2DL3, KIR2DL5A, KLRC1, KLRD1, PTPN6, PTPN11, ICAM1, ICAM2, ITGAL, ITGB2, PTK2B, VAV3, VAV1, VAV2, RAC1, RAC2, RAC3, PAK1, MAP2K1, MAP2K2, MAPK1, MAPK3, TNF, CSF2, IFNG, KIR2DS1, KIR2DS3, KIR2DS4, KIR2DS5, KIR2DS2, KLRC2, KLRC3, NCR2, TYROBP, LCK, IGH, FCGR3A, FCGR3B, NCR1, NCR3, FCER1G, CD247, ZAP70, SYK, LCP2, LAT, PLCG1, PLCG2, SH3BP2, PIK3CA, PIK3CD, PIK3CB, PIK3R1, PIK3R2, PIK3R3, FYN, SHC1, SHC2, SHC3, SHC4, GRB2, SOS1, SOS2, HRAS, KRAS, NRAS, ARAF, BRAF, RAF1, MICB, MICA, ULBP1, ULBP2, ULBP3, KLRK1, KLRC4-KLRK1, HCST, CD48, CD244, PPP3CA, PPP3CB, PPP3CC, PPP3R1, PPP3R2, NFATC1, NFATC2, PRKCA, PRKCB, PRKCG, SH2D1B, SH2D1A, IFNGR1, IFNGR2, IFNA1, IFNA2, IFNA4, IFNA5, IFNA6, IFNA7, IFNA8, IFNA10, IFNA13, IFNA14, IFNA16, IFNA17, IFNA21, IFNB1, IFNAR1, IFNAR2, TNFSF10, TNFRSF10A, TNFRSF10B, FASLG, FAS, GZMB, PRF1, CASP3, BID, RAET1G, RAET1L, RAET1E*
Antigen processing and presentation	*IFNG, TNF, PSME1, PSME2, PSME3, HSPA8, HSPA1A, HSPA2, HSPA1L, HSPA1B, HSPA6, HSPA4, HSP90AA1, HSP90AB1, HLA-A, HLA-B, HLA-C, HLA-F, HLA-G, HLA-E, HSPA5, CANX, B2M, PDIA3, CALR, TAPBP, TAP1, TAP2, CD8A, CD8B, CD8B2, KIR3DL2, KIR3DL1, KIR3DL3, KIR2DL2, KIR2DL1, KIR2DL3, KIR2DL4, KIR2DL5A, KLRC1, KLRC2, KLRC3, KLRC4, KLRD1, KIR2DS1, KIR2DS3, KIR2DS4, KIR2DS5, KIR2DS2, IFI30, LGMN, CTSB, HLA-DMA, HLA-DMB, HLA-DOA, HLA-DOB, HLA-DPA1, HLA-DPB1, HLA-DQA1, HLA-DQA2, HLA-DQB1, HLA-DRA, HLA-DRB1, HLA-DRB3, HLA-DRB4, HLA-DRB5, CD74, CTSL, CTSS, CD4, CIITA, RFX5, RFXANK, RFXAP, CREB1, NFYA, NFYB, NFYC*
T cell receptor signaling	*CD3D, CD3E, CD3G, CD247, CD4, CD8A, CD8B, CD8B2, PTPRC, LCK, FYN, ZAP70, LCP2, LAT, ITK, TEC, NCK1, NCK2, VAV3, VAV1, VAV2, GRAP2, GRB2, PAK1, PAK2, PAK3, PAK4, PAK5, PAK6, BUB1B-PAK6, RHOA, CDC42, DLG1, MAPK11, MAPK12, MAPK13, MAPK14, PLCG1, PPP3CA, PPP3CB, PPP3CC, PPP3R1, PPP3R2, NFATC1, NFATC2, NFATC3, SOS1, SOS2, RASGRP1, HRAS, KRAS, NRAS, RAF1, MAP2K1, MAP2K2, MAPK1, MAPK3, FOS, JUN, PRKCQ, CARD11, BCL10, MALT1, MAP3K7, MAP2K7, MAPK8, MAPK10, MAPK9, CHUK, IKBKB, IKBKG, NFKB1, RELA, NFKBIA, NFKBIB, NFKBIE, CD28, ICOS, CD40LG, PIK3R1, PIK3R2, PIK3R3, PIK3CA, PIK3CD, PIK3CB, PDPK1, AKT1, AKT2, AKT3, MAP3K8, MAP3K14, GSK3B, PDCD1, CTLA4, PTPN6, CBLB, IL2, IL4, IL5, IL10, IFNG, CSF2, TNF, CDK4*
B cell receptor signaling	*IGH, CD79A, CD79B, LYN, SYK, BTK, DAPP1, BLNK, VAV3, VAV1, VAV2, RAC1, RAC2, RAC3, PLCG2, PPP3CA, PPP3CB, PPP3CC, PPP3R1, PPP3R2, NFATC1, NFATC2, NFATC3, GRB2, SOS1, SOS2, RASGRP3, HRAS, KRAS, NRAS, RAF1, MAP2K1, MAP2K2, MAPK1, MAPK3, FOS, JUN, PRKCB, CARD11, BCL10, MALT1, CHUK, IKBKB, IKBKG, NFKB1, RELA, NFKBIA, NFKBIB, NFKBIE, IFITM1, CD81, CD19, CR2, PIK3R1, PIK3R2, PIK3R3, PIK3CA, PIK3CD, PIK3CB, AKT1, AKT2, AKT3, GSK3B, FCGR2B, INPP5D, INPPL1, LILRB2, LILRB1, LILRB5, LILRB4, LILRA1, LILRB3, LILRA3, LILRA2, LILRA4, LILRA6, LILRA5, LOC102725035, CD22, CD72, PTPN6, PIK3AP1*
Fc gamma R-mediated phagocytosis	*IGH, FCGR1A, FCGR2A, FCGR3A, FCGR3B, PTPRC, HCK, LYN, SYK, PIK3CA, PIK3CD, PIK3CB, PIK3R1, PIK3R2, PIK3R3, AKT1, AKT2, AKT3, RPS6KB1, RPS6KB2, PLCG1, PLCG2, PRKCD, PRKCE, RAF1, MAP2K1, MAPK1, MAPK3, PLA2G4E, PLA2G4A, JMJD7-PLA2G4B, PLA2G4B, PLA2G4C, PLA2G4D, PLA2G4F, PLA2G6, MARCKS, MARCKSL1, PLD1, PLD2, PLPP1, PLPP3, PLPP2, SPHK1, SPHK2, PRKCA, PRKCB, PRKCG, NCF1, GSN, SCIN, VAV3, VAV1, VAV2, CDC42, WAS, VASP, ARPC5, ARPC5L, ARPC4, ARPC3, ARPC1B, ARPC1A, ARPC2, RAC1, RAC2, WASF1, WASF2, WASF3, PAK1, LIMK1, LIMK2, CFL1, CFL2, PIP5K1C, PIP5K1A, PIP5K1B, ARF6, CRK, CRKL, DOCK1, ASAP1, ASAP3, ASAP2, FCGR2B, INPP5D, INPPL1, GAB2, LAT, DNM2, AMPH, BIN1, MYO10*
ECM-receptor interaction	*COL1A1, COL1A2, COL2A1, COL4A2, COL4A4, COL4A6, COL4A1, COL4A5, COL4A3, COL6A1, COL6A2, COL6A3, COL6A6, COL6A5, COL9A1, COL9A2, COL9A3, LAMA1, LAMA2, LAMA3, LAMA5, LAMA4, LAMB1, LAMB2, LAMB3, LAMB4, LAMC1, LAMC2, LAMC3, CHAD, RELN, THBS1, COMP, THBS2, THBS3, THBS4, FN1, SPP1, VTN, TNC, TNN, TNR, TNXB, NPNT, FRAS1, FREM2, FREM1, DSPP, VWF, IBSP, DMP1, AGRN, HSPG2, ITGA1, ITGA2, ITGA2B, ITGA3, ITGA4, ITGA5, ITGA6, ITGA7, ITGA8, ITGA9, ITGA10, ITGA11, ITGAV, ITGB1, ITGB3, ITGB4, ITGB5, ITGB6, ITGB7, ITGB8, CD44, SDC1, SDC4, SV2C, SV2B, SV2A, CD36, GP5, GP1BA, GP1BB, GP9, GP6, DAG1, CD47, HMMR*
Focal adhesion	*COL1A1, COL1A2, COL2A1, COL4A2, COL4A4, COL4A6, COL4A1, COL4A5, COL4A3, COL6A1, COL6A2, COL6A3, COL6A6, COL6A5, COL9A1, COL9A2, COL9A3, LAMA1, LAMA2, LAMA3, LAMA5, LAMA4, LAMB1, LAMB2, LAMB3, LAMB4, LAMC1, LAMC2, LAMC3, CHAD, RELN, THBS1, COMP, THBS2, THBS3, THBS4, FN1, SPP1, VTN, TNC, TNN, TNR, TNXB, VWF, IBSP, ITGA1, ITGA2, ITGA2B, ITGA3, ITGA4, ITGA5, ITGA6, ITGA7, ITGA8, ITGA9, ITGA10, ITGA11, ITGAV, ITGB1, ITGB3, ITGB4, ITGB5, ITGB6, ITGB7, ITGB8, PDGFA, PDGFB, PDGFC, PDGFD, EGF, IGF1, VEGFA, VEGFB, PGF, VEGFC, VEGFD, HGF, PDGFRA, PDGFRB, IGF1R, KDR, EGFR, FLT1, FLT4, MET, ERBB2, SRC, ARHGAP35, ARHGAP5, RHOA, DIAPH1, ROCK1, ROCK2, MYL2, MYL5, MYL7, MYL9, MYL10, MYL12B, MYL12A, MYLPF, PPP1CA, PPP1CB, PPP1CC, PPP1R12A, PPP1R12B, PPP1R12C, MYLK, MYLK2, MYLK3, MYLK4, PIP5K1C, PIP5K1A, PIP5K1B, ACTG1, ACTB, RASGRF1, CAPN2, ACTN1, ACTN4, TLN1, TLN2, FLNA, FLNC, FLNB, PXN, ILK, ZYX, VASP, VCL, PARVB, PARVA, PARVG, PDPK1, AKT1,*

	AKT2, AKT3, GSK3B, CTNNB1, PRKCA, PRKCB, PRKCG, PTK2, PIK3CA, PIK3CD, PIK3CB, PIK3R1, PIK3R2, PIK3R3, PTEN, VAV3, VAV1, VAV2, RAC1, RAC2, RAC3, PAK1, PAK2, PAK3, PAK4, PAK5, PAK6, BUB1B-PAK6, CDC42, BCAR1, CRK, CRKL, DOCK1, RAPGEF1, RAP1A, RAP1B, MAPK8, MAPK10, MAPK9, JUN, BRAF, CAV1, CAV2, CAV3, FYN, SHC1, SHC2, SHC3, SHC4, GRB2, SOS1, SOS2, HRAS, RAF1, MAP2K1, MAPK1, MAPK3, ELK1, CCND1, CCND2, CCND3, BIRC2, BIRC3, XIAP, BAD, BCL2
Tight junction	*CRB3, CLDN4, CLDN3, CLDN7, CLDN19, CLDN16, CLDN14, CLDN15, CLDN17, CLDN20, CLDN11, CLDN18, CLDN22, CLDN5, CLDN10, CLDN8, CLDN6, CLDN2, CLDN1, CLDN9, CLDN23, CLDN34, CLDN25, CLDN24, OCLN, F11R, JAM2, JAM3, BVES, CDC42, PARD6A, PARD6G, PARD6B, MPP5, MPP4, TJP3, PATJ, MPDZ, PRKCZ, PRKCI, AMOT, AMOTL1, AMOTL2, ARHGAP17, RAC1, NF2, LLGL2, LLGL1, DLG1, SCRIB, PPP2CA, PPP2CB, PPP2R1B, PPP2R1A, PPP2R2A, PPP2R2B, PPP2R2C, PPP2R2D, PARD3, TIAM1, TJP1, TJAP1, DLG2, DLG3, NEDD4, NEDD4L, CGN, CGNL1, ARHGEF2, RHOA, GATA4, MARVELD3, MAP3K1, MAPK8, MAPK10, MAPK9, JUN, CD1A, CD1B, CD1C, CD1D, CD1E, CFTR, CDK4, YBX3, SYMPK, PCNA, CCND1, ERBB2, RUNX1, HSPA4, SLC9A3R1, EZR, RDX, MSN, PRKCE, ACTG1, ACTB, CACNA1D, MAP3K5, MAP2K7, SRC, CTTN, HCLS1, ACTR2, ACTR3B, ACTR3C, ACTR3, WHAMM, WAS, VASP, PRKACA, PRKACB, PRKACG, RAB13, ARHGEF18, ROCK1, ROCK2, MYL2, EPB41L4B, STK11, PRKAA1, PRKAA2, PRKAB1, PRKAB2, PRKAG1, PRKAG3, PRKAG2, MYH9, MYH10, MYH11, MYH14, MYL6B, MYL6, MYL9, MYL12B, MYL12A, IGSF5, MAGI1, SYNPO, ACTN1, ACTN4, MICALL2, RAB8A, RAB8B, RAPGEF6, RAP1A, ITGB1, AFDN, TJP2, RAPGEF2, RAP2C, MARVELD2, TUBA1B, TUBA4A, TUBA3C, TUBA1A, TUBA1C, TUBA8, TUBA3E, TUBA3D, TUBAL3*
p53 signaling	*ATM, CHEK2, ATR, CHEK1, GORAB, CDKN2A, MDM2, MDM4, TP53, CDKN1A, CCND1, CCND2, CCND3, CDK4, CDK6, CCNE1, CCNE2, CDK2, SFN, RPRM, CCNB1, CCNB2, CDK1, GADD45A, GADD45B, GADD45G, GTSE1, FAS, PIDD1, TNFRSF10A, TNFRSF10B, CASP8, BID, BAX, PMAIP1, BBC3, TP53AIP1, SIVA1, BCL2L1, BCL2, TP53I3, EI24, SHISA5, PERP, ZMAT3, SIAH1, CYCS, APAF1, CASP9, CASP3, AIFM2, IGFBP3, IGF1, SERPINE1, ADGRB1, CD82, THBS1, SERPINB5, DDB2, RRM2B, RRM2, SESN1, SESN3, SESN2, PTEN, TSC2, STEAP3, COP1, RCHY1, CCNG1, CCNG2, PPM1D, TP73*
Mismatch repair	*SSBP1, PMS2, MLH1, MSH6, MSH2, MSH3, MLH3, RFC1, RFC4, RFC2, RFC5, RFC3, PCNA, EXO1, RPA1, RPA2, RPA3, RPA4, POLD1, POLD2, POLD3, POLD4, LIG1*
Homologous recombination	*SSBP1, RAD50, MRE11, NBN, ATM, BRCA1, BARD1, RBBP8, BRIP1, TOPBP1, ABRAXAS1, UIMC1, BABAM1, BABAM2, BRCC3, PALB2, BRCA2, SEM1, SYCP3, RPA1, RPA2, RPA3, RPA4, RAD51, RAD52, RAD51B, RAD51C, RAD51D, XRCC2, XRCC3, RAD54L, RAD54B, POLD1, POLD2, POLD3, POLD4, BLM, TOP3A, TOP3B, MUS81, EME1*
PI3K-Akt signaling	*EGF, TGFA, EREG, AREG, FGF1, FGF2, FGF3, FGF4, FGF17, FGF6, FGF7, FGF8, FGF9, FGF10, FGF16, FGF5, FGF18, FGF20, FGF22, FGF19, FGF21, FGF23, NGF, BDNF, NTF3, NTF4, INS, IGF1, IGF2, PDGFA, PDGFB, PDGFC, PDGFD, CSF1, KITLG, FLT3LG, VEGFA, VEGFB, PGF, VEGFC, VEGFD, HGF, ANGPT1, ANGPT2, ANGPT4, EFNA1, EFNA2, EFNA3, EFNA4, EFNA5, EGFR, ERBB2, ERBB3, ERBB4, FGFR1, FGFR2, FGFR3, FGFR4, NGFR, NTRK1, NTRK2, INSR, IGF1R, PDGFRA, PDGFRB, CSF1R, KIT, FLT3, FLT1, FLT4, KDR, MET, TEK, EPHA2, GRB2, SOS1, SOS2, HRAS, KRAS, NRAS, RAF1, MAP2K1, MAP2K2, MAPK1, MAPK3, IRS1, TLR2, TLR4, RAC1, IGH, SYK, CD19, PIK3AP1, GH1, GH2, CSH1, CSH2, PRL, OSM, IL2, IL3, IL6, IL4, IL7, IFNA1, IFNA2, IFNA4, IFNA5, IFNA6, IFNA7, IFNA8, IFNA10, IFNA13, IFNA14, IFNA16, IFNA17, IFNA21, IFNB1, EPO, CSF3, GHR, PRLR, OSMR, IL2RA, IL2RB, IL2RG, IL3RA, IL6R, IL4R, IL7R, IFNAR1, IFNAR2, EPOR, CSF3R, JAK1, JAK2, JAK3, COL1A1, COL1A2, COL2A1, COL4A2, COL4A4, COL4A6, COL4A1, COL4A5, COL4A3, COL6A1, COL6A2, COL6A3, COL6A6, COL6A5, COL9A1, COL9A2, COL9A3, LAMA1, LAMA2, LAMA3, LAMA5, LAMA4, LAMB1, LAMB2, LAMB3, LAMB4, LAMC1, LAMC2, LAMC3, CHAD, RELN, THBS1, COMP, THBS2, THBS3, THBS4, FN1, SPP1, VTN, TNC, TNN, TNR, TNXB, VWF, IBSP, ITGA1, ITGA2, ITGA2B, ITGA3, ITGA4, ITGA5, ITGA6, ITGA7, ITGA8, ITGA9, ITGA10, ITGA11, ITGAV, ITGB1, ITGB3, ITGB4, ITGB5, ITGB6, ITGB7, ITGB8, PTK2, PIK3CA, PIK3CD, PIK3CB, PIK3R1, PIK3R2, PIK3R3, F2R, CHRM1, CHRM2, LPAR1, LPAR2, LPAR3, LPAR4, LPAR5, LPAR6, GNB1, GNB2, GNB3, GNB4, GNB5, GNG2, GNG3, GNG4, GNG5, GNG7, GNG8, GNG10, GNG11, GNG12, GNG13, GNGT1, GNGT2, PIK3CG, PIK3R5, PIK3R6, PDPK1, STK11, PRKAA1, PRKAA2, DDIT4, TSC1, TSC2, RHEB, MLST8, MTOR, RPTOR, EIF4EBP1, EIF4E, EIF4E2, EIF4E1B, RPS6KB1, RPS6KB2, EIF4B, RPS6, PRKCA, PKN1, PKN2, PKN3, SGK1, SGK2, SGK3, C8orf44-SGK3, AKT1, AKT2, AKT3, MAGI1, MAGI2, PTEN, THEM4, PPP2CA, PPP2CB, PPP2R1B, PPP2R1A, PPP2R2A, PPP2R2B, PPP2R2C, PPP2R2D, PPP2R3B, PPP2R3C, PPP2R3A, PPP2R5B, PPP2R5C, PPP2R5D, PPP2R5E, PPP2R5A, HSP90AA1, HSP90AB1, HSP90B1, CDC37, CRTC2, PHLPP1, PHLPP2, TCL1A, TCL1B, MTCP1, NOS3, BRCA1, GSK3B, GYS2, GYS1, PCK1, PCK2, G6PC, G6PC2, G6PC3, MYC, CCND1, CDKN1A, CDKN1B, CDK2, CDK4, CDK6, CCND2, CCND3, CCNE1, CCNE2, FOXO3, RBL2, FASLG, BCL2L11, YWHAZ, YWHAB, YWHAQ, YWHAE, YWHAH, YWHAG, BAD, BCL2L1, BCL2, CASP9, CREB1, ATF2, ATF4, CREB3, CREB3L1, CREB3L2, CREB3L3, CREB3L4, CREB5, ATF6B, MCL1, RXRA, NR4A1, IKBKG, CHUK, IKBKB, RELA, NFKB1, MYB, MDM2, TP53*

Wnt signaling	*PORCN, WNT1, WNT2, WNT2B, WNT3, WNT3A, WNT4, WNT5A, WNT5B, WNT6, WNT7A, WNT7B, WNT8A, WNT8B, WNT9A, WNT9B, WNT10B, WNT10A, WNT11, WNT16, CER1, NOTUM, WIF1, SERPINF1, SOST, DKK1, DKK2, DKK4, SFRP1, SFRP2, SFRP4, SFRP5, RSPO1, RSPO2, RSPO3, RSPO4, LGR4, LGR5, LGR6, RNF43, ZNRF3, FZD1, FZD7, FZD2, FZD3, FZD4, FZD5, FZD8, FZD6, FZD10, FZD9, LRP5, LRP6, BAMBI, CSNK1E, TPTEP2-CSNK1E, DVL3, DVL2, DVL1, FRAT1, FRAT2, CSNK2A1, CSNK2A2, CSNK2A3, CSNK2B, NKD1, NKD2, CXXC4, SENP2, GSK3B, CTNNB1, AXIN1, AXIN2, APC, APC2, CSNK1A1L, CSNK1A1, TCF7, TCF7L1, TCF7L2, LEF1, CTNNBIP1, CBY1, CHD8, SOX17, CTBP1, CTBP2, CTNND2, CREBBP, EP300, RUVBL1, SMAD4, SMAD3, MAP3K7, NLK, MYC, JUN, FOSL1, CCND1, CCND2, CCND3, CCN4, PPARD, MMP7, PSEN1, PRKACA, PRKACB, PRKACG, TP53, SIAH1, CACYBP, SKP1, TBL1X, TBL1Y, TBL1XR1, BTRC, FBXW11, CUL1, RBX1, GPC4, ROR1, ROR2, RYK, VANGL2, VANGL1, PRICKLE1, PRICKLE2, PRICKLE4, PRICKLE3, INVS, DAAM1, DAAM2, RHOA, ROCK2, RAC1, RAC2, RAC3, MAPK8, MAPK10, MAPK9, PLCB1, PLCB2, PLCB3, PLCB4, CAMK2A, CAMK2D, CAMK2B, CAMK2G, PPP3CA, PPP3CB, PPP3CC, PPP3R1, PPP3R2, PRKCA, PRKCB, PRKCG, NFATC1, NFATC2, NFATC3, NFATC4*
TGF-β signaling	*CHRD, NOG, NBL1, MICOS10-NBL1, GREM1, GREM2, THBS1, DCN, FMOD, LEFTY1, LEFTY2, FST, BMP2, BMP4, BMP6, INHBB, BMP5, BMP7, BMP8B, BMP8A, GDF5, GDF6, GDF7, AMH, THSD4, FBN1, LTBP1, TGFB1, TGFB2, TGFB3, INHBA, INHBC, INHBE, NODAL, NEO1, HJV, BMPR1A, BMPR1B, ACVR1, BMPR2, ACVR2A, RGMA, RGMB, AMHR2, TGFBR1, TGFBR2, ACVR1B, ACVR2B, ACVR1C, BAMBI, SMAD1, SMAD5, SMAD9, SMAD2, SMAD3, SMAD4, SMAD6, SMAD7, SMURF1, SMURF2, ZFYVE9, ZFYVE16, HAMP, ID1, ID2, ID3, ID4, RBL1, E2F4, E2F5, TFDP1, CREBBP, EP300, SP1, TGIF1, TGIF2, MYC, CDKN2B, PITX2, RBX1, CUL1, SKP1, MAPK1, MAPK3, IFNG, TNF, RHOA, ROCK1, PPP2R1B, PPP2R1A, PPP2CA, PPP2CB, RPS6KB1, RPS6KB2*
Cell cycle	*CCND1, CCND2, CCND3, CDK4, CDK6, RB1, RBL1, RBL2, ABL1, HDAC1, HDAC2, E2F1, E2F2, E2F3, E2F4, E2F5, TFDP1, TFDP2, GSK3B, TGFB1, TGFB2, TGFB3, SMAD2, SMAD3, SMAD4, MYC, ZBTB17, CDKN2A, CDKN2B, CDKN2C, CDKN2D, CDKN1B, CDKN1C, CDKN1A, CCNE1, CCNE2, CDK2, SKP1, CUL1, RBX1, SKP2, CCNA2, CCNA1, CDC6, CDC45, CDC7, DBF4, CDK1, CCNB1, CCNB2, CCNB3, CDC25B, CDC25C, YWHAZ, YWHAB, YWHAQ, YWHAE, YWHAH, YWHAG, PLK1, WEE1, WEE2, PKMYT1, CCNH, CDK7, ANAPC1, ANAPC2, CDC27, ANAPC4, ANAPC5, CDC16, ANAPC7, CDC23, ANAPC10, ANAPC11, CDC26, ANAPC13, CDC20, PTTG1, PTTG2, ESPL1, SMC1A, SMC1B, SMC3, STAG2, STAG1, RAD21, TTK, BUB1, BUB3, BUB1B, MAD1L1, MAD2L1, MAD2L2, FZR1, CDC14B, CDC14A, ATR, ATM, TP53, CHEK1, CHEK2, CREBBP, EP300, PRKDC, MDM2, GADD45A, GADD45B, GADD45G, PCNA, SFN, CDC25A, ORC1, ORC2, ORC3, ORC4, ORC5, ORC6, MCM2, MCM3, MCM4, MCM5, MCM6, MCM7*

Supplementary Table 6. The marker genes of immune signatures and biological processes.

Immune signatures and biological processes	Gene set
Cytolytic activity [1]	*GZMA, PRF1*
Pro-inflammatory cytokines [2]	*IFNG, IL1A, IL1B, IL2*
Anti-inflammatory cytokines [2]	*TGFB1, IL10, IL4, IL11*
M1 macrophages [3]	*FCGR1A, IDO1, SOCS1, CXCL10*
M2 macrophages [3]	*MRC1, TGM2, FCER2, CCL22*
T cell-inflamed gene expression profile [4]	*CCL5, CD27, CD274, CD276, CD8A, CMKLR1, CXCL9, CXCR6, HLA-DQA1, HLA-DRB1, HLA-E, IDO1, LAG3, NKG7, PDCD1LG2, PSMB10, STAT1, TIGIT*
EMT [5]	*CDH2, SNAI1, TGFB1, ZEB1, ZEB2*

References

[1] Rooney, M.S., et al., Molecular and genetic properties of tumors associated with local immune cytolytic activity. Cell, 2015. 160(1-2): p. 48-61.

[2] Davoli, T., et al., Tumor aneuploidy correlates with markers of immune evasion and with reduced response to immunotherapy. Science, 2017. 355(6322).

[3] Martinez, F.O. and S. Gordon, The M1 and M2 paradigm of macrophage activation: time for reassessment. F1000Prime Rep, 2014. 6: p. 13.

[4] Cristescu, R., et al., Pan-tumor genomic biomarkers for PD-1 checkpoint blockade-based immunotherapy. Science, 2018. 362(6411): p. 197-+.

[5] He, Y., et al., Classification of triple-negative breast cancers based on Immunogenomic profiling. J Exp Clin Cancer Res, 2018. 37(1): p. 327.
